# Hemodialysis biocompatibility mathematical models to predict the inflammatory biomarkers released in dialysis patients based on hemodialysis membrane characteristics and clinical practices

**DOI:** 10.1038/s41598-021-01660-1

**Published:** 2021-11-29

**Authors:** Amira Abdelrasoul, Heloisa Westphalen, Shaghayegh Saadati, Ahmed Shoker

**Affiliations:** 1 Department of Chemical and Biological Engineering, University of Saskatchewan, 57 Campus Drive, Saskatoon, SK S7N 5A9 Canada; 2 Division of Biomedical Engineering, University of Saskatchewan, 57 Campus Drive, Saskatoon, SK S7N 5A9 Canada; 3 Nephrology Division, College of Medicine, University of Saskatchewan, 107 Wiggins Rd, Saskatoon, SK S7N 5E5 Canada; 4 Saskatchewan Transplant Program, St. Paul’s Hospital, 1702 20th Street West, Saskatoon, SK S7M 0Z9 Canada

**Keywords:** Biochemistry, Computational biology and bioinformatics, Molecular biology, Biomarkers, Health care, Medical research, Nephrology, Chemistry, Engineering, Materials science, Mathematics and computing

## Abstract

Chronic kidney disease affects millions of people around the globe and many patients rely on hemodialysis (HD) to survive. HD is associated with undesired life-threatening side effects that are linked to membrane biocompatibility and clinical operating conditions. The present study develops a mathematical model to predict the inflammatory biomarkers released in HD patients based on membrane morphology, chemistry, and interaction affinity. Based on the morphological characteristics of two clinical-grade HD membrane modules (CTA and PAES-PVP) commonly used in Canadian hospitals, a molecular docking study, and the release of inflammatory cytokines during HD and in vitro incubation experiments, we develop five sets of equations that describe the concentration of eight biomarkers (serpin/antithrombin-III, properdin, C5a, 1L-1α, 1L-1β, C5b-9, IL6, vWF). The equations developed are functions of membrane properties (pore size, roughness, chemical composition, affinity to fibrinogen, and surface charge) and HD operating conditions (blood flow rate, Qb, and treatment time, t). We expand our model based on available clinical data and increase its range of applicability in terms of flow rate and treatment time. We also modify the original equations to expand their range of applicability in terms of membrane materials, allowing the prediction and validation of the inflammatory response of several clinical and synthesized membrane materials. Our affinity-based model solely relies on theoretical values of molecular docking, which can significantly reduce the experimental load related to the development of more biocompatible materials. Our model predictions agree with experimental clinical data and can guide the development of novel materials and support evidence-based membrane synthesis of HD membranes, reducing the need for trial-and-error approaches.

## Introduction

### Chronic kidney disease and hemodialysis challenges

Chronic kidney disease (CKD) affects a large portion of the world's population and leads to the gradual loss of renal function. In 2017, the global prevalence of CKD was 9.1%, and the disease caused 1.2 million deaths^1^. To compensate for the decline in renal function, renal replacement therapies (RRTs) are prescribed to remove toxins and excess fluids. An estimated 2.5 million people rely on RRTs and this number is projected to increase by 100% in the next 10 years^2^. Furthermore, early evidence related to the recent upsurge of coronavirus disease 2019 (COVID-19) points to a concerning increase in the number of people requiring RRTs due to associated acute kidney injury (AKI)^3,4^.

Hemodialysis (HD) is a common RRT. This membrane-based treatment is life-sustaining for many patients but is associated with acute side effects, life-threatening disorders, and chronic conditions^5,6^. As the patient's blood passes through the dialyzer, the contact between blood components and the membrane surface can trigger a multifaceted series of protein adsorption events in addition to activation of blood proteins, leukocytes, platelets, and thrombus formation^7^. The extent of these reactions has been associated with the biocompatibility of the HD membranes.

Biocompatibility refers to the endurance of living cells in the presence of foreign structures and it is related to multiple properties of the membrane material, such as its chemical structure and surface properties, as well as its blood compatibility^7,8^. Issues arising from bio-incompatibility have puzzled researchers for decades and extraordinary efforts have been devoted to investigating the physicochemical properties of membrane materials and developing more biocompatible membranes. The development of synthetic polymeric membranes was a significant advancement, replacing the highly bio-incompatible cellulosic membranes that have been associated with poor clinical outcomes of HD patients^9,10^.

The synthesis of novel membrane materials or improved membrane morphology is challenging and costly, and most materials available still provoke concerning blood activation reactions^11^. Surface modifications have been a common strategy, and promising results have been obtained^12–14^. Even though improvements have been observed, current membrane synthesis approaches are still based on trial-and-error, which is neither time-efficient nor cost-effective. Advances in computational methods such as molecular docking and molecular dynamics simulations (MDS) have advanced the understanding of blood-membrane interactions and the development of novel materials^15,16^. However, these tools cannot directly predict the inflammatory response upon contact between blood and the membrane material. In addition to the biocompatibility of the HD membrane materials, the inflammatory response experienced by HD patients is influenced by the HD operating conditions (Qb, dialysate flow rate—Qd, and t). Even though the strong influence of these parameters has been demonstrated in the literature, no consensus has been reached on the optimal settings for treatment. These parameters set the hydrodynamic conditions inside the membrane filtration unit and play a key role in dialysis adequacy and affect protein adsorption, other blood interactions, and ultimately the clinical outcome for the patient^10^. HD patients treated with higher Qb (above 250 mL/min) have improved dialysis adequacy. However, the increased shear stress experienced by the blood components increases the risk of cell rupture and mechanical hemolysis, which negatively affects the immune system^17^. On the other hand, patients treated with Qb below 250 mL/min have reduced removal of toxins and a higher risk for all-cause mortality^10^. Longer treatment times are associated with favourable outcomes and higher survival rates, but the treatment time can be constrained by economic factors and patient conditions. The challenge to researchers is to determine the optimal combination of membrane materials and operating conditions to better treat the over 2 million people worldwide that rely on HD to survive.

Our group has identified major challenges in membrane science and technology applied to biomedical applications, especially related to HD^8,10,18^. We conducted in-depth investigations on the clinical HD membranes used in Canadian hospitals to evaluate the influence of morphological and chemical properties on the inflammatory response experienced by HD patients^19,20^. After investigating molecular-level interactions using hydrophobic and hydrophilic membranes^21^, we have explored the enhancement of biocompatibility of polymeric HD membranes utilizing a novel zwitterionic coatings^16,22–24^. We have also investigated the effects of hydrodynamic conditions on protein adsorption^25^. Based on a clinical observational study, in vitro protein adsorption, and an innovative in situ synchrotron-based imaging technique, increased contact time was determined to enable more interaction, leading to more adsorption and release of inflammatory cytokines.

Clinical studies of HD are very limited in the field due to the potential risk to patients' health. Combined with the need to optimize operating conditions, modelling studies have attracted the interest of researchers^26–28^. Still, so far, current models cannot predict the inflammatory response expected in HD patients. The goal of this study is to develop a simple yet accurate model to better understand the factors and interactions that dictate the release of pro-inflammatory cytokines. The model provides a prediction of the inflammatory response as a function of different membrane properties (pore size, surface roughness, percentage of sulfur, affinity to FB, and surface charge) and HD operating conditions (Qb and t). The goal of the models is to guide the development of novel membrane materials while significantly reducing experimental work as well as trial-and-error treatment of patients and the potential for undesired side effects in clinical practice.

### Blood activation and induction of inflammatory biomarkers during HD

**Figure 1:**
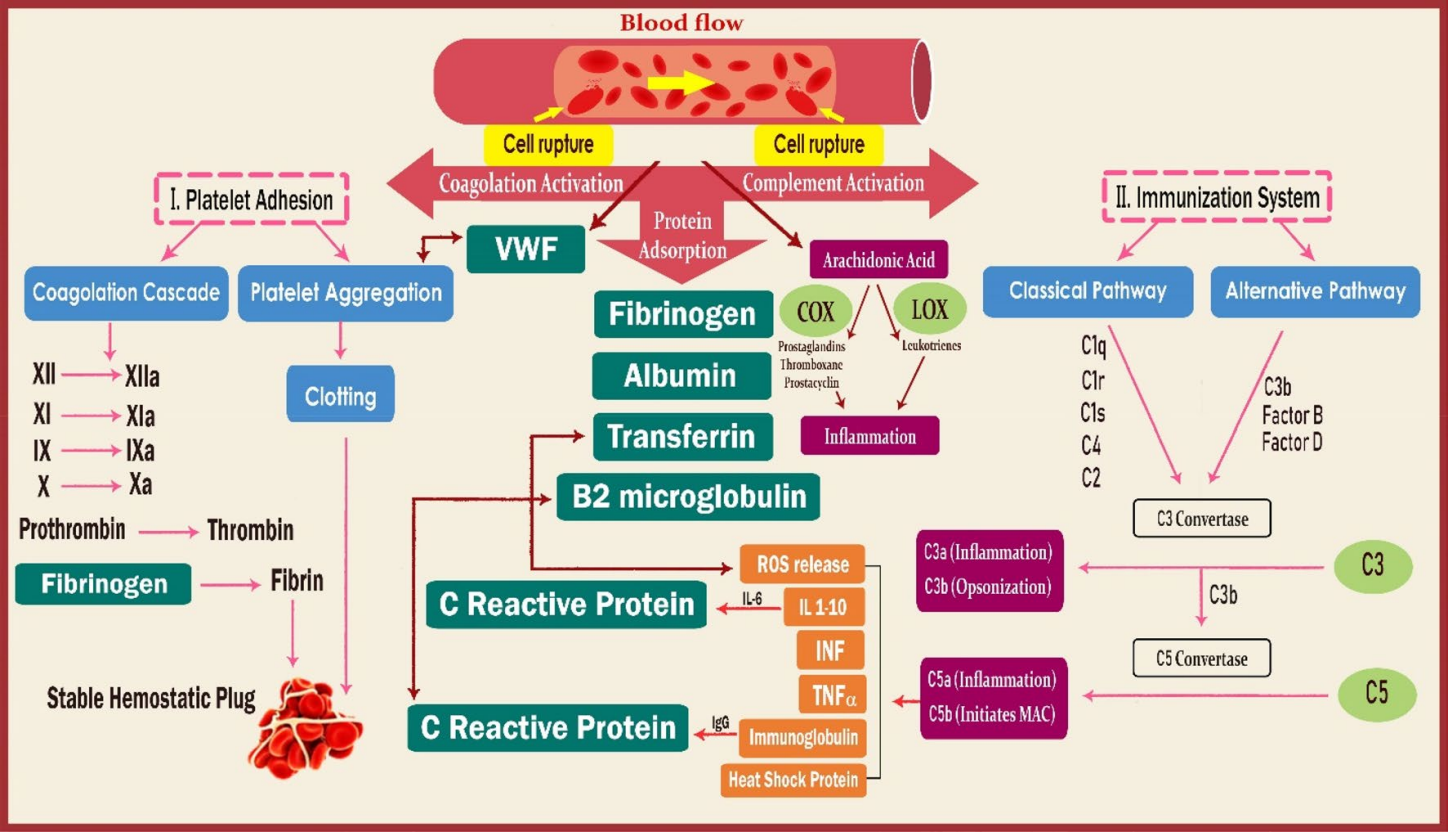
A schematic overview of key reactions occurring in blood and incited by the biomaterial surface. Protein adsorption is often accompanied by cell rupture, which initiates the complement system's intrinsic and extrinsic coagulation pathways. Coagulation pathway activation causes the production of a fibrin network. The figure includes complement activation and the appearance of complement proteins on the biomaterial interface. These represent the classical pathway as well as alternative pathway involving convertases that have been shown to participate in complement activation following response to the biomaterial. The scheme of eicosanoids biosynthesis pathways from arachidonic acid XII, XIIa, XI, Xia, IX, IXa, X and Xa: coagulation factors: vWF, von Willebrand factor, IL: interleukin, INF: interferon, IgG: immunoglobulin G, LOX: lipoxygenase, COX: cyclooxygenase.

Cell activation is initiated by blood exposure to HD membranes, and different biocompatibility leads to various blood activations (i.e., complement, coagulation, and thrombosis)^29^. The overall consequences of cell activations are not fully understood; however, chronic inflammation is known to be brought about by increases in various pro-inflammatory mediators and cytotoxic materials and ultimately involves the cardiovascular system^30,31^. High levels of inflammation are standard in patients undergoing HD due to interactions between the blood and dialyzer membrane, among other factors^32–35^. The inflammatory response involves the coagulation and complement systems, both of which take on essential functions on immunoprotection and hemostatic maintenance^31,32^. Contact of blood with foreign surfaces can initiate these types of cascades (Fig. 1). Both cascades feature factor X, which initiates prothrombin via a sequential series of factors leading to fibrin polymerization reaction in which FB plays an important role^33^. Because inflammation is part of the body's immune feedback, disrupted cells may likewise employ innate inflammatory cells whenever pathogens are absent^37^. Within the disrupted cells, arachidonic acid (AA) is changed by the COX-1 enzyme into thromboxane A2 (TXA2). This stimulates platelets through the thromboxane TP receptors^37^, and adenosine 5'-diphosphate (ADP) triggers the G protein-coupled receptors P2Y1 and P2Y12. The activation process using this specific receptor causes the entry of calcium from the extracellular compartment, respective shape change, and a transient collection of platelets (Fig. 1)^38^.

Complement activation can be triggered by antigen–antibody complexes as well as by bound C-reactive protein (CRP). Regardless of the pathway, complement activation leads to the cleavage of complement component 3 (C3). Increased C3b causes the generation of the C5-convertase and result in the production of C5a and C5b by cleavage of C5. C3a and C5a then bind to the HD membrane. Pro-inflammatory cytokines, such as interleukin (IL)-1β, tumor necrosis factor (TNF)-α, and IL-6, cause these reactions defined by the interaction between different receptors and ligands on the leukocytes. Activated leukocytes can release reactive oxygen species (ROS) that in turn can trigger platelets. ROS are overproduced in HD patients; specifically, 5-lipoxygenase (5-LO) catalyzes the oxygenation of AA to ROS formation^39^ and incites the release of lipoxins and leukotrienes. Activation of the 5-lipoxygenase pathways has been observed in uremia and HD^40^.

## Results

### Biocompatibility prediction model development

With the statistical analysis performed in this study, the concentration of each biomarker can be obtained using five different equations that produce the same result within the specified range of conditions. As five equations are obtained for the concentration of each of the eight biomarkers, a total of 40 equations are generated. These equations are in terms of actual factors and are valid for the specified ranges. The membrane properties (Dp, Ra, %S, K, and ζ) are significant terms in the responses of IL-1α, IL-6, and C5b-9. The membrane properties have marginally significant effects on the responses of C5a, vWF, and IL-1β but improved the model prediction. The membrane properties have an insignificant effect on the prediction models for serpin and properdin. The main effects and interactions are illustrated in Fig. S4(A–L), which shows the change in concentration (pg/mL) of each biomarker with the change in levels of each coded factor (instead of actual values, it shows the change from − 1 to 1). Having five sets of equations relating to different membrane properties and operating conditions increases the range of applicability of the equations. For instance, if estimating the inflammatory response using a membrane whose pore size is out of range is desired, another property such as roughness or zeta potential can be used to estimate the concentration of a specific biomarker. The fit statistics shown in Table S1 indicate the models accurately describe the data and present a signal-to-noise ratio greater than 4, indicating adequate precision.

### Biocompatibility prediction model based on membrane morphology (Dp and Ra)

Equations (1–8) (Box 1) show the concentration of C5a, properdin, C5b-9, serpin, IL-1β, IL-1α, IL-6, and vWF as a function of the operating conditions and Dp. Equations (9–16) (Box 2) show the same response but as a function of the operating conditions and Ra. These models indicate that as Dp or Ra are increased, the concentrations of C5a and IL-1β decrease and the concentration of vWF increases. For the concentration of IL-1α, significant interaction effects were identified. In Eqs. (6) and (14), the terms $(\mathrm{Dp}\,\times\,\mathrm{t})$ and $(\text{Ra}\,\times\,\text{t})$ indicate the changes in concentration of IL-1α caused by a change in Dp or Ra depends on the treatment time (Fig. S4G). This demonstrates that serum samples taken from all HD patients at the start of the HD session (t = 0 min) had similar concentrations of IL-1α; however, higher concentrations of IL-1α were found in serum samples that interacted for 30 min with smaller pore size membranes vs. larger pore size membranes or smoother membranes vs. rougher membranes. The $\mathrm{Dp}\,\times\,\mathrm{Qb}$ and $\mathrm{Ra}\,\times\,\mathrm{Qb}$ interactions have a significant negative effect on IL-6 (Fig. S4H) and positive effect on C5b-9 (Fig. S4I). This shows that, in the absence of flow (Qb = 0), levels of IL-6 and C5b-9 are not significantly different for the different membranes. However, patients treated with rougher (or larger Dp) membranes experience lower levels of IL-6 and higher levels of C5b-9 after hydrodynamic conditions are applied (Qb > 0).

### Biocompatibility prediction model based on membrane chemistry (%S and ${\varvec{\upzeta}}$)

The concentrations of C5a, properdin, C5b-9, serpin, IL-1β, IL-1α, IL-6, and vWF as a function of the %S and operating conditions are represented in Eqs. (17–24) (Box 3). Likewise, these responses are shown as a function of Qb, t and $\upzeta$ in Eqs. (25–32) (Box 4). Similar to what was observed for the morphological properties (Dp and Ra), the %S has a negative effect on the concentrations of C5a and IL-1β and a positive effect on vWF (Fig. S4A–C). Conversely, the opposite trends were observed in the $\upzeta$ models (Fig. S4D–F). The concentration of IL-1α is also significantly affected by the interaction terms $\mathrm{\%S}\,\times\,\mathrm{time}$ and $\upzeta\,\times\,\mathrm{time}$. Whereas concentrations of IL-6 and C5b-9 were significantly affected by the $\mathrm{\%S}\,\times\,\mathrm{Qb}$ and $\upzeta \times \mathrm{Qb}$ interaction terms (Fig. S4G–L).

### Biocompatibility prediction model based on membrane affinity to FB interactions

The concentrations of C5a, properdin, C5b-9, serpin, IL-1β, IL-1α, IL-6, and vWF as a function of the theoretical membrane affinity to fibrinogen (K) and operating conditions are represented in Eqs. (33–40) (Box 5). The models indicate that higher C5a and IL-1β are experienced as the membrane affinity to FB increases (becomes less negative). On the other hand, the concentration of vWF decreases as K increases (Fig. S4J–L). For IL-1α, the significant interaction identified is $\mathrm{K}\,\times\,\mathrm{time}$, which indicates the changes in response caused by a change in K depends on the treatment time. After 30 min of contact between blood and membrane, serum samples exposed to the membrane of higher K experience higher levels of IL-1α. The $\mathrm{K}\,\times\,\mathrm{Qb}$ interaction has a significant positive effect on IL-6 and a negative effect on C5b-9. This shows that, in the absence of flow (Qb = 0), levels of IL-6 and C5b-9 are not significantly different when different membranes are used. As hydrodynamic conditions are applied (Qb > 0), patients treated with lower (more negative) K membranes present lower levels of IL-6 and higher levels of C5b-9 within 30 min.

### Biocompatibility prediction model based on clinical practices (blood flow rate and dialysis treatment time)

The Qb is one of the most influential parameters in our models for the concentration of inflammatory biomarkers. Additionally, treatment time (which corresponds to the duration of the interaction between blood and the HD membrane) also influences the inflammatory response of HD patients. Qb has a positive effect on the concentration of all complement activation markers (C5a, properdin, and C5b-9). No interactions with other factors were identified for C5a, which means an increase in C5a concentration occurs as the Qb increases. Additionally, for a fixed Qb, the concentration of C5a decreases as the time of contact increases. Qb is a factor involved in interactions related to the concentrations of properdin and C5b-9. Specifically, the interaction between Qb and treatment time ($\mathrm{Qb }[\mathrm{mL}/\mathrm{min}]\cdot \mathrm{t }\left[\mathrm{min}\right]$). As the blood-membrane contact time increases, the concentrations of properdin and C5b-9 increase when Qb > 0, but it decreases in the absence of flow. In this case, the concentrations of properdin and C5b-9 decrease when Qb = 0 mL/min due to reduced shear effect, limited contact between blood and the membrane, and protein adsorption into the membrane structure. On the other hand, the concentration of properdin and C5b-9 increases when Qb > 0 mL/min. With more contact promoted by increased flow, more severe complement activation is observed.

The hydrodynamic conditions strongly influence the concentration of serpin. Qb has a positive impact and treatment time has a negative effect, and these factors have significant interaction. Upon interaction between blood and membrane, the concentration of serpin decreases in both the absence of flow and under hydrodynamic conditions, but at a much higher rate for the latter. This behaviour is associated with serpin being a coagulation and clotting factor.

The concentration of vWF is not significantly influenced by treatment time but increases with increasing Qb. The concentration of IL-6 decreases with increasing time, which can also be associated with adsorption to the membrane or removal via convective transport. Qb is involved in an interaction with membrane properties in equations for the concentration of IL-6. For the concentrations of IL-1β and IL-1α, Qb is involved in an interaction with time. As the contact time increases, the concentration of IL-1β decreases in the absence of flow and increases under hydrodynamic conditions. The concentration of IL-1α increases slightly when Qb > 0 and increases at a much higher rate in the absence of flow. IL-1α concentration is also influenced by the interaction between time and membrane properties, which means the change in the concentration of this biomarker over time depends on the membrane properties. The model trend indicates that reducing the zeta potential (more negative) would result in a decrease in the concentration of IL-1α experienced by HD patients at 30 min.

## Expanded biocompatibility prediction model

### Expanded model for blood flow rate and HD treatment time

The basic statistical model was expanded based on historical data of HD patients to increase the applicability of the original equations. To that end, correction factors (${\varphi }_{E}$) (Table 1) were calculated for each factor and can be added to respective sets of equations. For instance, the affinity-based model represented by Eqs. (33–40) (Box 5) was modified to Eqs. (41–48) (Box 6), and the same can be done to any of the models presented in section “Predicted vs. Experimental Values of Inflammatory Biomarkers in HD Patients”.

**Table 1:** Hydrodynamic correction factors (${\mathrm{\varphi }}_{\mathrm{E}}$) for expanded model for CTA and PAES-PVP membranes.

Expanded operating conditions		Hydrodynamic correction factor (${\varphi }_{E}$)							
t (min)	Qb (mL/min)	C5a	IL-1β	IL-1α	IL-6	vWF	Serpin	Properdin	C5b-9
30	200 < Qb ≤ 300	1.39E + 05	0.01	3.38	13.735	0	1.75E + 06	− 4.90E + 06	3.64E + 03
30	300 < Qb ≤ 400	− 2.89E + 04	− 0.32	1.08	8.04	− 561.26	2.45E + 08	− 4.28E + 06	− 3.64E + 03
30	400 < Qb < 500	− 2.89E + 04	− 0.32	1.08	10.05	− 883.98	− 3.94E + 07	− 1.22E + 07	− 2.28E + 03
90	200 < Qb ≤ 300	4.62E + 04	− 0.5015	0.58328	0.737	− 175.3925	3.63E + 08	− 1.18E + 07	− 2.28E + 03
90	300 < Qb ≤ 400	3.47E + 03	− 0.89	2.54	9.38	− 420.94	6.13E + 08	− 1.47E + 07	− 8.19E + 03
90	400 < Qb ≤ 500	3.47E + 03	− 0.89	2.16	12.06	− 561.26	7.01E + 08	− 2.26E + 07	− 7.74E + 03
240	200 < Qb ≤ 300	4.62E + 04	− 1.18	2.0288	1.608	− 175.3925	1.66E + 09	− 3.67E + 07	− 9.10E + 03
240	300 < Qb ≤ 400	9.24E + 04	− 2.66	3.93	14.07	− 1.40	1.82E + 09	− 5.45E + 07	− 1.87E + 04
240	400 < Qb < 500	9.24E + 04	− 4.13	6.34	14.07	− 456.02	2.36E + 09	− 7.96E + 07	− 2.23E + 04

**Figure 2:**
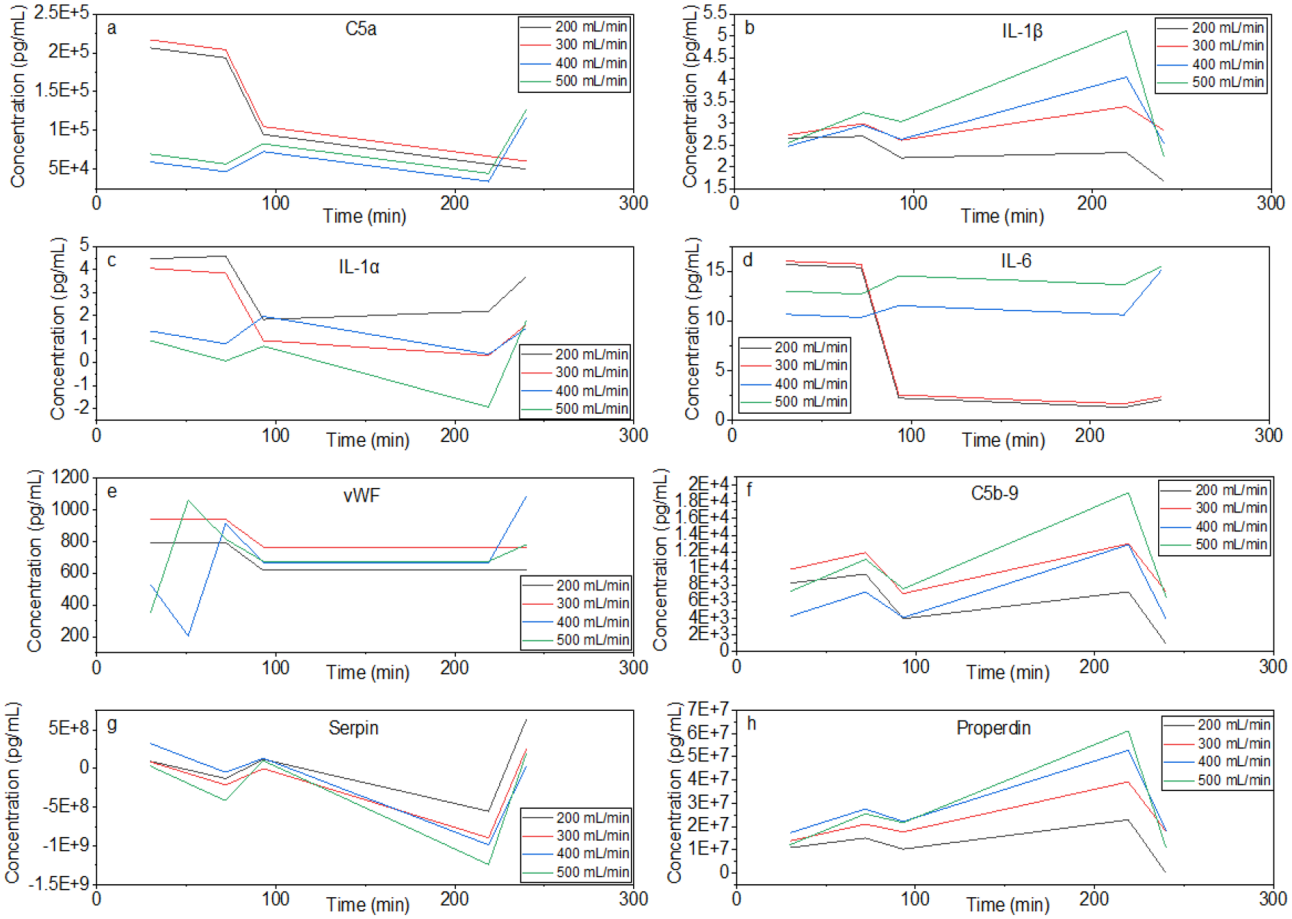
Prediction of biomarker concentrations using the extended model (Origin(Pro), Version 2020b, https://www.originlab.com/index.aspx?go=Products/Origin).

For this expanded model, the Qb range is extended up to 500 mL/min and t to 240 min. The equations are applicable for the following ranges of membrane properties: 0.851 nm < Dp < 8.24 nm, 5.4 nm < Ra < 10.4 nm, − 68 mV < ζ <  − 34 mV, 0 < %S < 3.83, and − 6 kcal/mol < K <  − 5.3 kcal/mol. This model can be used to predict the inflammatory response of HD patients treated with CTA and PAES-PVP dialyzers. The correction factors should be chosen from Table 1, according to the desired time and Qb. When using the expanded model, predictions are made for flow rates and treatment times above zero. By choosing a value of zero for the correction factor, the original model is restored. The predicted trends for the PAES-PVP membrane can be found in Fig. 2. According to the prediction model developed in this study, HD treatment using PAES-PVP membranes should result in higher C5a, C5b-9, IL-1α, IL-6, and vWF within the first 30 min in patients treated with lower Qb. This corroborates with lower flow rates promoting slower and more pronounced adsorption of FB, leading to more severe inflammatory and thrombotic responses.

### Expanded model for various clinical membrane materials

The original developed models were then expended for a wider variety of materials based on the incubation of uremic blood with different membrane materials. Membrane properties of PAN, PVDF, PVDF-ZW, and PES-ZW were obtained using the same methodology described for CTA and PAES membranes. Based on %S, affinity to FB, roughness, zeta potential, and pore size of these materials, new correction factors (${\varphi }_{M}$) were calculated for specific ranges as seen in Table 2. The new correction factors were added to the original models (Boxes 1, 2, 3, 4, and 5). For instance, the affinity-based model in Box 5 becomes the new model in Box 7 (Eqs. 49–56). The affinity to FB (K) of different membrane materials (CTA, PAES, PAN, PVDF, PVDF-ZW) was obtained using molecular docking (Fig. S3), with values listed in Table S2. Importantly, these expanded models for different materials were developed based on incubation experiments, and therefore the equations must be evaluated at Qb = 0 and t = 30 min. When using material correction factors (${\varphi }_{M}$), the equations predict the release of biomarkers in the absence of flow. The two correction factors cannot be used in combination due to the distinct nature of each one.

**Table 2:** Material correction factors (${\varphi }_{M}$) for various clinical membrane materials.

Membrane Properties					φ_m_*						
%S	K (kcal/mol)	Ra (nm)	ζ (mV)	Dp (nm)	C5a	IL-1β	IL-1α	IL-6	Serpin	Properdin	C5b-9
0 ≤ %S < 0.12	− 5.2 ≥ K > − 5.6	5.4 ≤ Ra ≤ 12	− 41.5 < ζ ≤ − 2.5	0.851 ≤ dp ≤ 8	9.24E + 04	2.36	3.38	11.39	1.75E + 06	− 4.90E + 06	2503.04
	− 5.6 ≥ K ≥ − 6	9 < Ra ≤ 10	− 42 ≤ ζ ≤ − 41.5	6 ≥ dp ≥ 8	1.16E + 05	0.27	3.38	12.06	1.75E + 06	− 6.12E + 06	1137.75
0.12 ≤ %S < 3.83	− 6 > K ≥ − 6.7	9 < Ra ≤ 10	− 45 < ζ ≤ − 42	6 ≤ dp < 8	1.39E + 05	0.01	3.38	13.74	1.75E + 06	− 4.90E + 06	3640.79
%S ≥ 3.83	− 5.6 ≥ K ≥ − 6	9 < Ra ≤ 10.4	− 68 ≤ ζ ≤ − 45	7 < dp ≤ 8.24	1.16E + 05	0.27	3.38	12.06	1.75E + 06	− 6.12E + 06	1137.75

*These correction factors can be added to any of the respective biomarker equations while setting Qb = 0 and t = 30 min. For C5a, the factors can be added to Eqs. (1, 9, 17, 25, and 33); for IL-1β, the factors can be added to Eqs. (5, 13, 21, 29, and 37); for IL-1α, the factors can be added to Eqs. (6, 14, 22, 30, and 36); for IL-6, the factors can be added to Eqs. (7, 15, 23, 31, and 39); for serpin, the factors can be added to Eqs. (4, 12, 20, 28, and 36); for properdin, the factors can be added to Eqs. (2, 10, 18, 26, and 34); and for C5b-9, the factors can be added to Eqs. (3, 11, 19, 27, and 35).

## Biocompatibility model validation

### Predicted vs. experimental values of inflammatory biomarkers in HD patients

**Figure 3:**
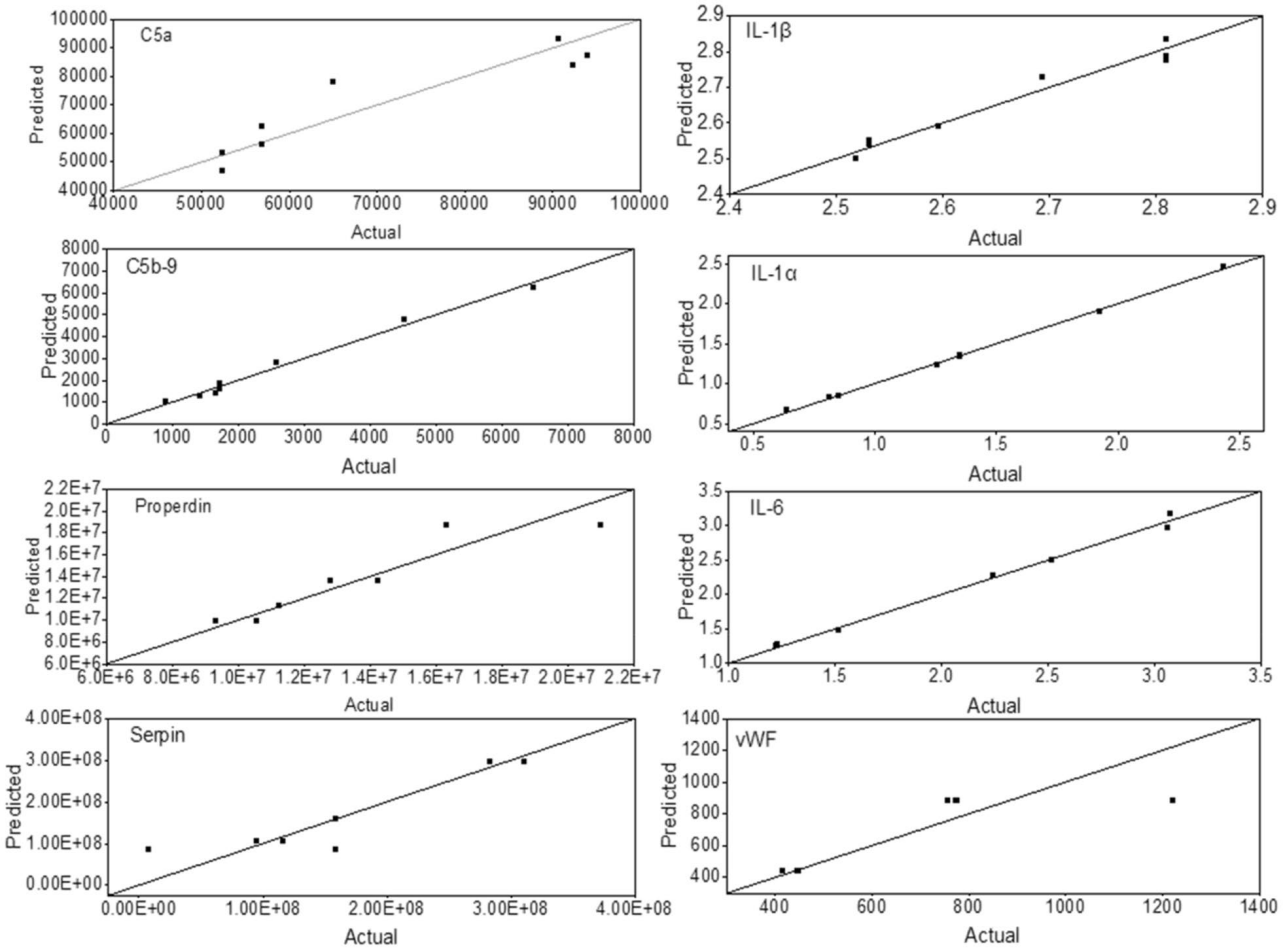
Predicted vs. actual values of the concentration (pg/mL) of C5a, IL-1β, C5b-9, IL-1α, properdin, IL-6, serpin, and vWF. Predicted values were obtained using Eqs. (33–40) (affinity-based model) without correction factors (Origin(Pro), Version 2020b, https://www.originlab.com/index.aspx?go=Products/Origin).

Equations (1)–(40) were validated by comparing the predicted results to actual experimental values. The actual values were obtained from serum samples collected pre-dialysis and 30 min after the beginning of the HD session and from serum samples incubated with CTA and PAES-PVP membranes for 30 min. Figure 3 shows the predicted vs. actual value plots indicating good agreement between the data. The predicted values were obtained from Eqs. (33–40) (Box 5, affinity-based model without any correction factors); however, the predicted value of each biomarker can be calculated with any of the models presented (i.e., based on affinity, pore size, roughness, etc.) and the response is the same. While utilizing membrane properties other than CTA and PAES-PVP, minor differences might be observed between different sets of equations. It is recommended to consider the average response of the different sets of equations and frame the lower and upper bounds of the expected response interval in terms of the lowest and highest responses.

### Prediction of inflammatory biomarker release in HD patients

To further validate our models, we compared the predicted values for the concentration of all biomarkers with the concentrations measured in serum samples collected at 30, 90, and 240 min of HD sessions using CTA and PAES-PVP membranes. The percent error was calculated for each case and showed good agreement between the predicted and actual values. The predicted values presented in Table 3 were obtained utilizing the affinity-based model (Eqs. 41–48, Box 6) with the hydrodynamic correction factors shown in Table 1. The predicted responses moderately agree with historical patient data, with percent errors ranging from 0.5% to over 100% in some cases. The model is more accurate when predicting the concentration of most biomarkers at 30 and 90 min, with greater error at higher flow rates. For times beyond 30 min, the model underpredicts the concentration of serpin with high percent errors. Similarly, for times over 90 min, the concentration of properdin is overpredicted with large discrepancies. However, the concentration of these biomarkers in pg/mL is on the order of 10^7^–10^8^, so even acceptable discrepancies lead to high % error.

**Table 3:** Predicted [obtained from Eqs. (41–48), and correction factor from Table 1] and actual values for the concentrations of all biomarkers based on the expanded model for CTA and PAES-PVP membranes.

Qb (mL/min)	Time (min)	Membrane	C5a			IL-1β			IL-1α			IL-6		
			Predicted*	Actual	%error	Predicted*	Actual	%error	Predicted*	Actual	%error	Predicted*	Actual	%error
300	30	CTA	8.39E + 04	9.23E + 04	9.1	2.8	2.8	1.3	1.2	1.3	1.8	3.0	3.1	3.3
300	30	CTA	8.39E + 04	4.92E + 04	70.3	2.8	3.0	7.1	1.2	1.1	16.3	3.0	1.2	148.5
300	30	CTA	8.39E + 04	1.35E + 05	38.0	2.8	2.6	5.2	1.2	1.5	15.0	3.0	4.9	40.0
300	30	PAES	7.79E + 04	6.49E + 04	20.0	2.7	2.7	1.1	0.7	0.6	4.8	2.3	2.2	1.3
300	30	PAES	7.79E + 04	8.00E + 04	2.6	2.7	2.8	2.4	0.7	0.6	4.8	2.3	1.4	57.6
300	30	PAES	7.79E + 04	4.98E + 04	56.3	2.7	2.6	4.9	0.7	0.6	4.8	2.3	3.0	25.3
200	90	PAES	4.94E + 04	1.40E + 04	253.4	2.7	2.5	9.6	1.2	0.6	95.0	1.5	2.9	48.4
300	90	CTA	6.56E + 04	1.07E + 05	38.8	3.1	2.8	13.1	2.0	1.4	39.4	2.5	2.9	12.7
300	90	PAES	5.97E + 04	8.84E + 04	32.5	3.1	2.9	8.3	0.4	1.0	61.6	1.8	2.6	29.1
400	90	PAES	6.99E + 04	8.38E + 04	16.5	3.5	2.5	39.1	− 0.5	2.3	122.7	2.2	10.5	79.3
500	90	PAES	8.02E + 04	8.92E + 04	10.1	3.9	2.8	40.1	− 1.4	0.9	265.0	2.5	12.6	80.1
200	240	PAES	3.76E + 03	1.00E + 05	96.2	2.8	2.5	12.4	1.7	2.5	32.4	0.4	13.0	96.9
300	240	PAES	1.40E + 04	6.93E + 04	79.7	4.0	2.7	49.6	− 0.4	1.6	125.3	0.7	2.3	68.4
300	240	CTA	2.00E + 04	9.85E + 04	79.7	4.1	2.4	72.2	4.0	2.5	57.8	1.4	2.9	51.5
400	240	PAES	2.43E + 04	1.28E + 05	81.0	5.2	2.6	101.9	− 2.5	1.5	270.3	1.1	13.0	91.8
500	240	PAES	3.46E + 04	1.08E + 05	68.0	6.3	2.1	208.3	− 4.5	1.8	350.7	1.4	13.2	89.3
			vWF			Serpin			Properdin			C5b-9		
Qb	Time	Membrane	Predicted*	Actual	%error	Predicted*	Actual	%error	Predicted*	Actual	%error	Predicted*	Actual	%error
300	30	CTA	824.4	776.5	6.2	8.38E + 07	1.59E + 08	47.3	1.87E + 07	2.10E + 07	11.1	2834.8	2580.1	9.9
300	30	CTA	824.4	201.8	308.5	8.38E + 07	7.28E + 06	1050.5	1.87E + 07	1.29E + 07	44.8	2834.8	1254.1	126.1
300	30	CTA	824.4	1351.1	39.0	8.38E + 07	3.11E + 08	73.1	1.87E + 07	2.91E + 07	35.9	2834.8	3906.1	27.4
300	30	PAES	940.2	757.4	24.1	8.38E + 07	8.47E + 06	888.7	1.87E + 07	1.63E + 07	14.3	6223.3	6478.1	3.9
300	30	PAES	940.2	480.3	95.8	8.38E + 07	1.25E + 07	572.5	1.87E + 07	2.00E + 07	6.7	6223.3	5998.9	3.7
300	30	PAES	940.2	1034.5	9.1	8.38E + 07	4.49E + 06	1766.3	1.87E + 07	1.27E + 07	47.6	6223.3	6957.3	10.5
200	90	PAES	792.4	365.0	117.1	− 2.28E + 08	1.07E + 08	313.5	2.18E + 07	9.14E + 06	138.1	6126.0	4073.5	50.4
300	90	CTA	824.4	939.8	12.3	− 3.42E + 08	4.67E + 08	173.3	2.90E + 07	1.74E + 07	66.6	8819.3	2901.4	204.0
300	90	PAES	940.2	775.6	21.2	− 3.42E + 08	2.11E + 07	1718.2	2.90E + 07	1.71E + 07	69.3	9085.6	8207.3	10.7
400	90	PAES	1087.9	681.3	59.7	− 4.56E + 08	1.18E + 08	485.8	3.61E + 07	1.54E + 07	134.4	12,045.2	3851.6	212.7
500	90	PAES	1235.7	569.3	117.0	− 5.69E + 08	8.96E + 07	735.3	4.33E + 07	1.71E + 07	152.7	15,004.7	7019.8	113.8
300	240	CTA	824.4	828.4	0.5	− 1.41E + 09	5.71E + 08	346.2	5.46E + 07	2.05E + 07	166.4	15,975.0	2310.3	591.5
300	240	PAES	940.2	692.1	35.8	− 1.41E + 09	2.65E + 08	630.4	5.46E + 07	1.81E + 07	202.3	16,241.3	6681.4	143.1
200	240	PAES	792.4	1388.9	42.9	− 1.03E + 09	7.59E + 08	235.3	3.67E + 07	2.15E + 07	71.0	9983.2	7020.7	42.2
400	240	PAES	1087.9	1290.4	15.7	− 1.79E + 09	3.39E + 07	5363.9	7.25E + 07	1.35E + 07	438.0	22,499.5	3801.4	491.9
500	240	PAES	1235.7	1263.2	2.2	− 2.17E + 09	1.45E + 08	1597.6	9.04E + 07	1.18E + 07	666.1	28,757.7	6575.3	337.4

*Predicted values obtained with the affinity-based model (Eqs. 41–48). Similar results are obtained if any of the models are used within the experimental range defined. The responses obtained using different models differ between 0 and 2% on average.

### Prediction of inflammatory biomarkers for different membrane materials using the biocompatibility models

We also validated our expanded model for various membrane materials. The properties of all membranes utilized in the study are summarized in Table S3. Using Eqs. (49–56) (Box 7) and the appropriate correction factors from Table 2, we predicted the concentration of seven biomarkers and compared them to actual values from incubation experiments. The comparison between actual and predicted values is shown in Table 4, with excellent agreement observed. The model is valid within the range of membrane properties, for flow rates between 0 and 300 mL/min and for contact times between 0 to 30 min. The average error for each biomarker ranges from 2.26 to 23%. Once again, any set of equations can be used to calculate the predicted values because the difference between the responses of different models is between 0 and 2% on average. These results support the assertion that our model can predict the release of inflammatory biomarkers as a function of membrane properties. In particular, our model can predict the concentration of seven important biomarkers utilizing the theoretical affinity to FB calculated using molecular docking studies.

**Table 4:** Predicted [obtained from Eqs. (49–56), and correction factors from Table 2] vs. actual for different materials.

Membrane	C5a			IL-1β			IL-1α			IL-6		
	Predicted*	Actual	Error%	Predicted*	Actual	Error%	Predicted*	Actual	Error%	Predicted*	Actual	Error%
PAES-PVP	4.71E + 04	5.24E + 04	10.17	2.50	2.52	0.71	1.90	1.93	1.38	1.26	1.23	2.69
CTA	5.31E + 04	5.23E + 04	1.41	2.55	2.53	0.70	2.46	2.43	1.09	1.26	1.22	3.12
PVDF	1.46E + 05	1.57E + 05	6.83	4.92	4.59	7.11	5.92	5.47	8.27	12.65	12.83	1.42
PAN	1.66E + 05	1.61E + 05	3.22	2.79	2.74	1.98	5.60	4.69	19.30	13.32	13.41	0.67
PES	1.63E + 05	1.66E + 05	1.78	2.77	3.36	17.66	5.28	6.34	16.79	13.32	12.62	5.57
PVDF-ZW	1.80E + 05	1.75E + 05	2.85	2.46	2.13	15.56	4.72	5.59	15.56	15.00	14.99	0.09

Membrane	Serpin			Properdin			C5b-9					
	Predicted*	Actual	Error%	Predicted*	Actual	Error%	Predicted*	Actual	Error%			
PAES-PVP	1.06E + 08	9.48E + 07	11.59	9.95E + 06	9.33E + 06	6.64	1302.88	1436.01	9.27			
CTA	1.06E + 08	1.17E + 08	9.41	9.95E + 06	1.06E + 07	5.86	1036.60	903.46	14.74			
PVDF	1.08E + 08	1.13E + 08	4.61	5.05E + 06	4.93E + 06	2.55	3501.60	3457.32	1.28			
PAN	1.08E + 08	8.73E + 07	23.27	3.83E + 06	3.80E + 06	0.71	2288.46	2571.55	11.01			
PES	1.08E + 08	2.95E + 08	63.49	3.83E + 06	5.92E + 06	35.34	2440.62	2146.03	13.73			
PVDF-ZW	1.08E + 08	1.44E + 08	25.55	5.05E + 06	7.18E + 06	29.57	5209.94	5011.32	3.96			

*Predicted values obtained with the affinity-based model (Eqs. 49–56). Similar results are obtained if any of the models are used within the experimental range defined. The responses obtained using different models differ between 0 and 2% on average.

## Discussion

For each biomarker, all five equations are in agreement; therefore, within the specified ranges, so the predicted response can be estimated according to membrane chemistry, morphology, or theoretical affinity to FB. The equations have R^2^ values ranging from 76.69 to 99.88%, indicating they accurately describe most responses. Our model based on pore size suggests smaller pore size membranes lead to higher levels of inflammatory cytokines. The adsorption of FB is also affected by pore size^20^. PAES membranes have slower but more pronounced adsorptive behaviour than CTA membranes, attributed to the larger pore size and surface area of the PAES membrane, allowing more irreversible attachment of proteins within the porous structure^25^. High levels of inflammatory cytokines and vWF are noted in patient serum during HD^41,42^. However, concurrent decreases in C5a and IL-1β are linked to the release of free radicals during complement cascade, leading to a significant decline in C5a and regulation of the production of IL-1β^43^.

Increased membrane roughness enhances the shear stress experienced by blood components and can lead to hemolysis and promote protein adsorption. According to the roughness-based model, the use of smoother vs. rougher membranes leads to significant increases in the concentrations of most biomarkers in only 30 min. Additionally, under flow conditions, the rougher membrane leads to a clear increase in the release of C5b-9 and vWF, which confirms the mechanical hemolysis due to the increased shear stress experienced near the membrane surface^44^.A rougher surface intensifies FB adsorption, which leads to more severe inflammatory response in HD patients^41,42^. Importantly, the Qb range investigated is on the lower side of the spectrum of prescribed Qb for Canadian and American patients (mean prescribed Qb of 371.85 and 425.42 mL/min^45^, respectively), so a more pronounced tendency for hemolysis can be expected for those patients, especially when rough membranes are used.

Regarding the membrane chemistry, our model indicates that lower %S in HD membranes leads to higher levels of C5a and IL-1β. However, the concentration of vWF also increased as the %S increased. Higher %S in HD membranes has been shown to aggravate FB adsorption, leading to increased levels of inflammatory cytokines and vWF in HD patients^20,42,43^. The presence of trace amounts of sulfur and nitrogen in the composition of the PAES-PVP membrane has been associated with decreased biocompatibility. Molecular dynamics simulation and docking studies have observed the role of sulfur interactions in protein adsorption^16,19^. The presence of sulfur has been shown to influence FB adsorption and the release of inflammatory biomarkers^20,24^. The CTA membrane has no sulfur in its composition but still causes activation of biochemical cascades; however, PAES membranes have been classified as a strong coagulation activator compared to CTA membranes.

Among the various physicochemical properties of membranes, surface charges and hydrophobicity play key roles in membrane-based bioeffects, including protein binding, oxidative stress, and inflammation. The higher surface charge presented by the PAES membrane makes it more hydrophilic, promoting the adsorption of water in the blood. The dehydration of RBCs can lead to accentuated hemolysis. Furthermore, the hydrophilicity of the membrane surface affects the adsorption of FB^46^. This is reflected in the increased levels of vWF observed when a more negative ζ membrane is used^41,42^. Some of the inflammatory biomarkers occur at lower concentrations even when the more hydrophilic membrane is used. This can be due to other membrane properties that control the separation of components via convective and diffusive mechanisms.

A material with a more negative K has a stronger affinity to FB and hence has a greater tendency to adsorb FB molecules. Protein adsorption is enhanced with greater affinity for FB, which leads to higher levels of inflammatory cytokines in addition to high levels of vWF in patient serum during HD^41,42^; the exception is levels of C5a and IL-1β, as discussed^43^. Notably, both membranes provoked the release of biomarkers, and the behaviour of both seems to be influenced by the hydrodynamic conditions. At Qb > 0, PAES-PVP membranes experience slower but greater adsorption of FB but are more likely to experience reversible and irreversible fouling as well as back-filtration^20,25^. This theoretical model (Box 5) is an innovative way to predicted response to the interaction between uremic serum and HD membranes. It can support evidence-based membrane synthesis to enable scientists to predict inflammatory responses based on the material's affinity; this represents a more time-efficient and cost-effective strategy and reduces the need for trial-and-error approaches.

The operating conditions of HD treatment influence the inflammatory response in different ways, depending on the membrane properties as seen in the interaction effects. In the absence of flow (Qb = 0), the concentrations of most biomarkers are lower than observed at Qb > 0. The mild results observed at Qb = 0 demonstrate how the hydrodynamic conditions influence the inflammatory response. This influence is manifested in different ways. First, only fixed amounts of blood components contact the membrane when Qb = 0, thus limiting contact-triggered reactions. Second, the pressure on the blood side increases when the flow is present, "pushing" the blood components towards the membrane surface and facilitating adsorption-mediated reactions. Third, the effect of shear stress is eliminated when Qb = 0, which reduces the rupture of RBCs, protein adsorption, and platelet adhesion. These results agree with recent reports which show that the FB adsorption is slower under hydrodynamic conditions (Qb > 0) but more pronounced for the PAES-PVP membrane, which is aligned with the theoretical model where the strong affinity for FB that leads to the more severe inflammatory response.

We expanded the models to broaden the applicability range to a Qb of 500 mL/min and a treatment time of 240 min. The predicted responses moderately agreed with historical patient data with percent errors ranging from 0.5 to > 100% in some cases. This expanded model can guide medical professionals to predict the concentration of eight biomarkers in patients treated with HD membranes with properties that fall within the studied range. Furthermore, we modified the equations to apply the model to a broader range of membrane properties. This modified model (Box 7) presented good agreement with experimental data and can hence guide the development of novel membrane materials for HD and other biomedical applications.

In summary, the models in Boxes 1, 3 and 4 (and their respective expanded models) are function of blood flow rate treatment time and can be easily used in clinical practice. The membrane properties (Dp, %S and ζ) are easy to measure and can be provided by the dialyzer manufacturer. Models in Boxes 2 and 5 (and their respective expanded models) are a function of Ra and K, which are not as simple to obtain, so its applicability is more suitable for membrane development. Although, our current models were based on a limited population size, but the current models are promising based on the validated results using different membranes and extra participants’ data. The developed models serve as a foundation for optimizing clinical practices in hemodialysis and membrane development. Our group will continue to devote our efforts to expand and develop robust models to strengthen its accuracy and generalize its applicability.

**Box 1:** Concentrations of biomarkers as a function of membrane pore size (Dp), Qb, and t.

$$C5a \left[ {pg/mL} \right] = 62887.95{ } - { }808.46 \cdot {\text{Dp }}\left[ {{\text{nm}}} \right]{ } + { }102.68 \cdot {\text{Qb }}\left[ {{\text{mL}}/{\text{min}}} \right] - 304.16 \cdot {\text{t }}\left[ {{\text{min}}} \right]$$	(1)
$$Properdin \left[ {pg/mL} \right] = 1.12{\text{E}}^{7} { } + { }7680.83 \cdot {\text{Qb }}\left[ {{\text{mL}}/{\text{min}}} \right]{ }{-}{ }42987.50 \cdot {\text{t }}\left[ {{\text{min}}} \right]{ } + { }713.90 \cdot {\text{Qb }}\left[ {{\text{mL}}/{\text{min}}} \right] \cdot {\text{t }}\left[ {{\text{min}}} \right]$$	(2)
$$C5b9 \left[ {{\text{pg}}/{\text{mL}}} \right] = 1553.94 + 36.04 \cdot {\text{Dp }}\left[ {{\text{nm}}} \right]{ }{-}1.80 \cdot {\text{Qb }}{-}18.27 \cdot {\text{t }}\left[ {{\text{min}}} \right] + 1.41 \cdot {\text{Dp }}\left[ {{\text{nm}}} \right] \cdot {\text{Qb }}\left[ {{\text{mL}}/{\text{min}}} \right] + 0.22 \cdot {\text{Qb }}\left[ {{\text{mL}}/{\text{min}}} \right] \cdot {\text{t }}\left[ {{\text{min}}} \right]$$	(3)
$${\text{Serpin}} \left[ {{\text{pg}}/{\text{mL}}} \right] = 1.59{\text{E}}^{8} { } + { }4.58{\text{E}}^{5} \cdot {\text{Qb }}\left[ {{\text{mL}}/{\text{min}}} \right] - { }1.78{\text{E}}^{6} \cdot {\text{t }}\left[ {{\text{min}}} \right]{ }{-}{ }17722.047 \cdot {\text{Qb }}\left[ {{\text{mL}}/{\text{min}}} \right] \cdot {\text{t }}\left[ {{\text{min}}} \right]$$	(4)
$$IL1\beta \left[ {{\text{pg}}/{\text{mL}}} \right] = 2.84 - 0.0066 \cdot {\text{Dp }}\left[ {{\text{nm}}} \right]{ }{-}0.00082 \cdot {\text{Qb }}{-}0.0095 \cdot {\text{t }}\left[ {{\text{min}}} \right] + 0.000052 \cdot {\text{Qb}} \cdot {\text{t }}\left[ {{\text{min}}} \right]$$	(5)
$$IL1\alpha \left[ {{\text{pg}}/{\text{mL}}} \right] = 1.36 - 0.0024 \cdot {\text{Dp }}\left[ {{\text{nm}}} \right]{ }{-}0.0017 \cdot {\text{Qb }}\left[ {{\text{mL}}/{\text{min}}} \right] + 0.039 \cdot {\text{t }}\left[ {{\text{min}}} \right] - 0.0024 \cdot {\text{Dp}} \cdot {\text{t }}\left[ {{\text{min}}} \right] - 7.9{\text{E}}^{5} \cdot {\text{Qb }}\left[ {{\text{mL}}/{\text{min}}} \right] \cdot {\text{t }}\left[ {{\text{min}}} \right]$$	(6)
$$IL6 \left[ {{\text{pg}}/{\text{mL}}} \right] = 1.48{ } + { }0.00068 \cdot {\text{Dp }}\left[ {{\text{nm}}} \right] + 0.005933 \cdot {\text{Qb }}\left[ {{\text{mL}}/{\text{min}}} \right] - 0.0073 \cdot {\text{t }}\left[ {{\text{min}}} \right] - 0.00031 \cdot {\text{Dp}} \cdot {\text{Qb }}\left[ {{\text{mL}}/{\text{min}}} \right]$$	(7)
$$vWF \left[ {{\text{pg}}/{\text{mL}}} \right] = 367.77 + 15.67 \cdot {\text{Dp }}\left[ {{\text{nm}}} \right] + 1.48 \cdot {\text{Qb }}\left[ {{\text{mL}}/{\text{min}}} \right]$$	(8)

**Box 2:** Concentrations of biomarkers as a function of membrane roughness (Ra), Qb, and t.

$$C5a = 68651.52{ }{-}{ }1194.74 \cdot {\text{Ra }}\left[ {{\text{nm}}} \right] + { }102.68 \cdot {\text{Qb }}\left[ {{\text{mL}}/{\text{min}}} \right] - 304.16 \cdot {\text{t }}\left[ {{\text{min}}} \right]$$	(9)
$$Properdin = 1.12{\text{E}}^{7} + 7680.83 \cdot {\text{Qb }}\left[ {{\text{mL}}/{\text{min}}} \right]{ }{-}{ }42987.50 \cdot {\text{t }}\left[ {{\text{min}}} \right] + 713.90 \cdot {\text{Qb }}\left[ {{\text{mL}}/{\text{min}}} \right]{ } \cdot {\text{t }}\left[ {{\text{min}}} \right]$$	(10)
$$C5b9 = 1297.03 + 53.25 \cdot {\text{Ra }}\left[ {{\text{nm}}} \right]{ }{-}11.84 \cdot {\text{Qb }}\left[ {{\text{mL}}/{\text{min}}} \right]{ }{-}18.27 \cdot {\text{t }}\left[ {{\text{min}}} \right] + 2.08 \cdot {\text{Ra }}\left[ {{\text{nm}}} \right] \cdot {\text{Qb }}\left[ {{\text{mL}}/{\text{min}}} \right]{ } + 0.22 \cdot {\text{Qb }}\left[ {{\text{mL}}/{\text{min}}} \right]{ } \cdot {\text{t }}\left[ {{\text{min}}} \right]$$	(11)
$$Serpin = 1.59{\text{E}}^{8} + 4.58{\text{E}}^{5} \cdot {\text{Qb }}\left[ {{\text{mL}}/{\text{min}}} \right]{ } - 1.78{\text{E}}^{6} \cdot {\text{t }}\left[ {{\text{min}}} \right]{ }{-}17722.05 \cdot {\text{Qb }}\left[ {{\text{mL}}/{\text{min}}} \right] \cdot {\text{t }}\left[ {{\text{min}}} \right]$$	(12)
$$IL1\beta = 2.89 - 0.0097 \cdot {\text{Ra }}\left[ {{\text{nm}}} \right]{ }{-}0.00082 \cdot {\text{Qb }}\left[ {{\text{mL}}/{\text{min}}} \right] - 0.0095 \cdot {\text{t }}\left[ {{\text{min}}} \right] + 0.000052 \cdot {\text{Qb }}\left[ {{\text{mL}}/{\text{min}}} \right] \cdot {\text{t }}\left[ {{\text{min}}} \right]$$	(13)
$$IL1\alpha = 1.38 - 0.0035 \cdot {\text{Ra }}\left[ {{\text{nm}}} \right]{ }{-}0.0017 \cdot {\text{Qb }}\left[ {{\text{mL}}/{\text{min}}} \right] + 0.056 \cdot {\text{t }}\left[ {{\text{min}}} \right] - 0.0036 \cdot {\text{Ra }}\left[ {{\text{nm}}} \right] \cdot {\text{t }}\left[ {{\text{min}}} \right] - 7.9{\text{E}}^{5} \cdot {\text{Qb }}\left[ {{\text{mL}}/{\text{min}}} \right] \cdot {\text{t }}\left[ {{\text{min}}} \right]$$	(14)
$$IL6 = 1.47 + 0.0010 \cdot {\text{Ra }}\left[ {{\text{nm}}} \right] + 0.0082 \cdot {\text{Qb }}\left[ {{\text{mL}}/{\text{min}}} \right] - 0.0073 \cdot {\text{t }}\left[ {{\text{min}}} \right]{ } - { }0.00046 \cdot {\text{Ra }}\left[ {{\text{nm}}} \right] \cdot {\text{Qb }}\left[ {{\text{mL}}/{\text{min}}} \right]$$	(15)
$$vWF = 256.04 + 23.16 \cdot {\text{Ra }}\left[ {{\text{nm}}} \right] + 1.48 \cdot {\text{Qb }}\left[ {{\text{mL}}/{\text{min}}} \right]$$	(16)

**Box 3:** Concentrations of biomarkers as a function of membrane percentage of sulfur (%S), Qb, and t.

$$C5a = 62199.95 - 1559.71 \cdot {\text{\% S}} + 102.68 \cdot {\text{Qb }}\left[ {{\text{mL}}/{\text{min}}} \right] - 304.16 \cdot {\text{t }}\left[ {{\text{min}}} \right]$$	(17)
$$Properdin = 1.125{\text{E}}^{7} + 7680.83 \cdot {\text{Qb }}\left[ {{\text{mL}}/{\text{min}}} \right]{ }{-}{ }42987.50 \cdot {\text{t }}\left[ {{\text{min}}} \right] + 713.90 \cdot {\text{Qb }}\left[ {{\text{mL}}/{\text{min}}} \right] \cdot {\text{t }}\left[ {{\text{min}}} \right]$$	(18)
$$C5b9 = 1584.61{ } + 69.52 \cdot {\text{\% S}} - 0.60 \cdot {\text{Qb }}\left[ {{\text{mL}}/{\text{min}}} \right] - 18.26696 \cdot {\text{t }}\left[ {{\text{min}}} \right] + { }2.72 \times {\text{\% S}} \cdot {\text{Qb }}\left[ {{\text{mL}}/{\text{min}}} \right]{ } + 0.22 \cdot {\text{Qb }}\left[ {{\text{mL}}/{\text{min}}} \right] \cdot {\text{t }}\left[ {{\text{min}}} \right]$$	(19)
$$Serpin = 1.59{\text{E}}^{8} + 4.58{\text{E}}^{5} \cdot {\text{Qb }}\left[ {{\text{mL}}/{\text{min}}} \right] - 1.78{\text{E}}^{6} \cdot {\text{t }}\left[ {{\text{min}}} \right]{-}17722.05 \cdot {\text{Qb }}\left[ {{\text{mL}}/{\text{min}}} \right] \cdot {\text{t }}\left[ {{\text{min}}} \right]$$	(20)
$$IL1\beta = 2.83 - 0.013 \cdot {\text{\% S}} - 0.00082 \cdot {\text{Qb }}\left[ {{\text{mL}}/{\text{min}}} \right] - 0.0095 \cdot {\text{t }}\left[ {{\text{min}}} \right] + 0.000052 \cdot {\text{Qb }}\left[ {{\text{mL}}/{\text{min}}} \right] \cdot {\text{t }}\left[ {{\text{min}}} \right]$$	(21)
$$IL1\alpha = 1.36{ } - { }0.0046 \cdot {\text{\% S}} - 0.0017 \cdot {\text{Qb }}\left[ {{\text{mL}}/{\text{min}}} \right] + 0.036741 \cdot {\text{t }}\left[ {{\text{min}}} \right] - 0.0047 \cdot {\text{\% S}} \cdot {\text{t }}\left[ {{\text{min}}} \right] - 7.9{\text{E}}^{5} \cdot {\text{Qb }}\left[ {{\text{mL}}/{\text{min}}} \right] \cdot {\text{t }}\left[ {{\text{min}}} \right]$$	(22)
$$IL6 = 1.37 + 0.0013 \cdot {\text{\% S}} + 0.0057 \cdot {\text{Qb }}\left[ {{\text{mL}}/{\text{min}}} \right] - 0.000603 \cdot {\text{\% S}} \cdot {\text{Qb }}\left[ {{\text{mL}}/{\text{min}}} \right]{ }$$	(23)
$$vWF = 381.11 + 30.24 \cdot {\text{\% S}} + 1.48 \cdot {\text{Qb }}\left[ {{\text{mL}}/{\text{min}}} \right]$$	(24)

**Box 4:** Concentrations of biomarkers as a function of membrane zeta potential ($\upzeta$), Qb, and t.

$$C5a = 68173.63 - 175.70 \cdot {\zeta }\left[ {{\text{mV}}} \right] + 102.68 \cdot {\text{Qb }}\left[ {{\text{mL}}/{\text{min}}} \right] - 304.16 \cdot {\text{t }}\left[ {{\text{min}}} \right]$$	(25)
$$Properdin = 1.12{\text{E}}^{7} + 7680.83 \cdot {\text{Qb }}\left[ {{\text{mL}}/{\text{min}}} \right]{ }{-}{ }42987.50 \cdot {\text{t }}\left[ {{\text{min}}} \right] + 713.90 \cdot {\text{Qb }}\left[ {{\text{mL}}/{\text{min}}} \right] \cdot {\text{t }}\left[ {{\text{min}}} \right]$$	(26)
$$C5b9 = 1318.33 - 7.83 \cdot {\zeta }\left[ {{\text{mV}}} \right] - 11.01 \cdot {\text{Qb }}\left[ {{\text{mL}}/{\text{min}}} \right] - 18.27 \cdot {\text{t }}\left[ {{\text{min}}} \right] - 0.31 \cdot {\zeta }\left[ {{\text{mV}}} \right] \cdot {\text{Qb }}\left[ {{\text{mL}}/{\text{min}}} \right] + 0.22 \cdot {\text{Qb }}\left[ {{\text{mL}}/{\text{min}}} \right] \cdot {\text{t }}\left[ {{\text{min}}} \right]$$	(27)
$Serpin = 1.59{\text{E}}^{8} + 4.58{\text{E}}^{5} \cdot {\text{Qb }}\left[ {{\text{mL}}/{\text{min}}} \right] - 1.78{\text{E}}^{6} \cdot {\text{t }}\left[ {{\text{min}}} \right]{-}17722.05 \cdot {\text{Qb }}\left[ {{\text{mL}}/{\text{min}}} \right] \cdot {\text{t }}\left[ {{\text{min}}} \right]$.	(28)
$$IL1\beta = 2.88 - 0.0014 \cdot {\zeta }\left[ {{\text{mV}}} \right] - 0.00082 \cdot {\text{Qb }}\left[ {{\text{mL}}/{\text{min}}} \right] - 0.0095 \cdot {\text{t }}\left[ {{\text{min}}} \right] + 0.000052 \cdot {\text{Qb }}\left[ {{\text{mL}}/{\text{min}}} \right] \cdot {\text{t }}\left[ {{\text{min}}} \right]$$	(29)
$IL1\alpha = 1.38 + { }0.000515 \cdot {\zeta }\left[ {{\text{mV}}} \right] - 0.001725 \cdot {\text{Qb }}\left[ {{\text{mL}}/{\text{min}}} \right] + 0.055 \cdot {\text{t }}\left[ {{\text{min}}} \right] + 0.000534{ } \cdot {\zeta }\left[ {{\text{mV}}} \right] \cdot {\text{t }}\left[ {{\text{min}}} \right] - 7.9{\text{E}}^{5} \cdot {\text{Qb }}\left[ {{\text{mL}}/{\text{min}}} \right] \cdot {\text{t }}\left[ {{\text{min}}} \right]$.	(30)
$$IL6 = 1.47 + 0.00015 \cdot {\zeta }\left[ {{\text{mV}}} \right] + 0.007975 \cdot {\text{Qb }}\left[ {{\text{mL}}/{\text{min}}} \right] - 0.007292 \cdot {\text{t }}\left[ {{\text{min}}} \right] + 6.8{\text{E}}^{5} \cdot {\zeta }\left[ {{\text{mV}}} \right] \cdot {\text{Qb }}\left[ {{\text{mL}}/{\text{min}}} \right]{ }$$	(31)
$$vWF = 265.30 - 3.41 \cdot {\zeta }\left[ {{\text{mV}}} \right] + 1.48 \cdot {\text{Qb }}\left[ {{\text{mL}}/{\text{min}}} \right]$$	(32)

**Box 5:** Concentrations of biomarkers as a function of membrane affinity to fibrinogen (K), Qb, and t.

$$C5a = 1.074{\text{E}}^{5} - 8533.82 \cdot {\text{K }}\left[ {{\text{kcal}}/{\text{mol}}} \right] + 102.68 \cdot {\text{Qb }}\left[ {{\text{mL}}/{\text{min}}} \right] - 304.16 \cdot {\text{t }}\left[ {{\text{min}}} \right]$$	(33)
$$Properdin = 1.12{\text{E}}^{7} + 7680.83 \cdot {\text{Qb }}\left[ {{\text{mL}}/{\text{min}}} \right]{ }{-}{ }42987.50 \cdot {\text{t }}\left[ {{\text{min}}} \right] + 713.90 \cdot {\text{Qb }}\left[ {{\text{mL}}/{\text{min}}} \right] \cdot {\text{t }}\left[ {{\text{min}}} \right]$$	(34)
$$C5b9 = - 431.49 - 380.40 \cdot {\text{K}}\left[ {{\text{kcal}}/{\text{mol}}} \right] - 79.40 \cdot {\text{Qb}}\left[ {{\text{mL}}/{\text{min}}} \right] - 18.27 \cdot {\text{t}}\left[ {{\text{min}}} \right] - 14.87 \cdot {\text{K }}\left[ {{\text{kcal}}/{\text{mol}}} \right] \cdot {\text{Qb }}\left[ {{\text{mL}}/{\text{min}}} \right] + 0.22 \cdot {\text{Qb}}\left[ {{\text{mL}}/{\text{min}}} \right] \cdot {\text{t}}\left[ {{\text{min}}} \right]$$	(35)
$$Serpin = 1.59{\text{E}}^{8} + 4.58{\text{E}}^{5} \cdot {\text{Qb }}\left[ {{\text{mL}}/{\text{min}}} \right] - 1.78{\text{E}}^{6} \cdot {\text{t }}\left[ {{\text{min}}} \right]{-}17722.05 \cdot {\text{Qb }}\left[ {{\text{mL}}/{\text{min}}} \right] \cdot {\text{t }}\left[ {{\text{min}}} \right]$$	(36)
$$IL1\beta = 3.20 + 0.069 \cdot {\text{K }}\left[ {{\text{kcal}}/{\text{mol}}} \right] - 0.00082 \cdot {\text{Qb }}\left[ {{\text{mL}}/{\text{min}}} \right] - 0.009472{ } \cdot {\text{t }}\left[ {{\text{min}}} \right] + { }0.000052{ } \cdot {\text{Qb }}\left[ {{\text{mL}}/{\text{min}}} \right]{ } \cdot {\text{t }}\left[ {{\text{min}}} \right]$$	(37)
$$IL1\alpha = 1.49 + 0.025 \cdot {\text{K }}\left[ {{\text{kcal}}/{\text{mol}}} \right] - 0.0017 \cdot {\text{Qb }}\left[ {{\text{mL}}/{\text{min}}} \right] + 0.17 \cdot {\text{t }}\left[ {{\text{min}}} \right] + 0.026 \cdot {\text{K }}\left[ {{\text{kcal}}/{\text{mol}}} \right] \cdot {\text{t }}\left[ {{\text{min}}} \right] - 7.9{\text{E}}^{5} \cdot {\text{Qb }}\left[ {{\text{mL}}/{\text{min}}} \right] \cdot {\text{t }}\left[ {{\text{min}}} \right]$$	(38)
$$IL6 = 1.44 - 0.0071 \cdot {\text{K }}\left[ {{\text{kcal}}/{\text{mol}}} \right] + 0.023 \cdot {\text{Qb }}\left[ {{\text{mL}}/{\text{min}}} \right] - 0.0073 \cdot {\text{t }}\left[ {{\text{min}}} \right] + 0.0033 \cdot {\text{K }}\left[ {{\text{kcal}}/{\text{mol}}} \right] \cdot {\text{Qb }}\left[ {{\text{mL}}/{\text{min}}} \right]$$	(39)
$$vWF = - 495.72 - 165.44 \cdot {\text{K }}\left[ {{\text{kcal}}/{\text{mol}}} \right] + 1.48 \cdot {\text{Qb }}\left[ {{\text{mL}}/{\text{min}}} \right]$$	(40)

## Materials and methods

### Characterization of HD clinical membrane chemistry and morphology

We obtained actual clinical membrane modules utilized in Canadian hospitals, comprised of either CTA (Exeltra 210 dialyzer) or a blended PAES-PVP polymer (also referred to herein as simply PAES) (REVACLEAR 400 dialyzer)^20^. These medical-grade membranes were supplied by St. Paul's Hospital, Saskatoon, Canada, and are recognized in the medical field for their optimal filtration flux, solute removal, and hemocompatibility.

Atomic force microscopy (AFM) micrographs (Fig. S1) show clearly distinct morphological characteristics of CTA and PAES HD membrane fibers^20^. The CTA images show prominent fibrous strains indicated as lined strokes, whereas the internal and external features of the PAES fibers are distinctly porous and stretched/lined. The porous side could make up a sectioned separation layer with an active pathway for diffusive transport of solutes. Corresponding magnitudes of average roughness (Ra) and root mean square roughness (RRMS) are summarized in Table S4. Both membrane materials present similar roughness on both sides of their fibers. However, the Ra values of the PAES membrane (10.4 ± 4.0 nm for the inside) were greater than for the CTA membrane (5.4 ± 1.9 nm).

We used x-ray photoelectron spectroscopy (XPS) analyses to determine the surface chemistry of both sides of the fibers^20^. The spectra for respective sample surfaces were analyzed with the aid of the CasaXPS software as presented in Fig. S2. The spectra of each side of each material show similar peaks with no discernable differences due to similarities in chemistry; however, notable differences were evident when comparing the CTA and PAES membranes. The percentage abundance of elements within each membrane fiber is shown in Table S5. These results indicate the CTA membrane is mainly composed of C and O, with less C on the outer side of the fiber. The PAES membrane fibers also contain high percentages of C and O in addition to less than 4% S and N, which indicate a low blending ratio between PAES and PVP.

Our zeta potential and Brunauer–Emmett–Teller (BET) analyses show the surface charge of the PAES-PVP membrane (− 64 mV) is approximately double that of the CTA membrane (− 38 mV) (Table S6). The polymeric membrane presents a broader pore size distribution compared to the cellulosic membrane. The measured pore size for the polymeric and cellulosic membranes is 8.24 and 0.85 nm, respectively^20^.

### Clinical study of inflammatory biomarkers

A cohort of 12 HD patients using PAES-PVP and CTA dialyzers from the St. Paul's Hospital dialysis center and two healthy controls were recruited. The inclusion criteria were classification as either normal (control) or having kidney disease (HD patients), male or female, and under 60 years of age. Basic patient information can be found in Table S7. All participants’ vascular access consisted of arteriovenous (AV) fistula. The HD patients were prescribed different Qb values that ranged between 200 and 500 mL/min. All patients were prescribed a dialysate flow rate (Qd) of 500 mL/min and a treatment time of 4 h. Following ethical approval of the study, blood samples were collected from all patients at the beginning of the HD session, at different times during the session (30 and 90 min), and at the end of the session (240 min). The blood samples were prepared and analyzed for the presence of serpin/antithrombin-III, properdin, C5a, IL-1α, IL-1β, IL-6, the Von Willebrand factor (vWF), and terminal complement complex (C5b-9), utilizing Human Magnetic Lumix Assays as described in our previous study^20^. All samples and controls were measured in triplicate.

**Box 6:** Expanded model for blood flow rate and treatment time ranges for the concentrations of biomarkers as a function of membrane affinity to fibrinogen (K), Qb, and t.

$$C5a = 1.074{\text{E}}^{5} - 8533.82 \cdot {\text{K }}\left[ {{\text{kcal}}/{\text{mol}}} \right] + 102.68 \cdot {\text{Qb }}\left[ {{\text{mL}}/{\text{min}}} \right] - 304.16 \cdot {\text{t }}\left[ {{\text{min}}} \right] + \varphi_{E}$$	(41)
$$Properdin = 1.12{\text{E}}^{7} + 7680.83 \cdot {\text{Qb }}\left[ {{\text{mL}}/{\text{min}}} \right]{ }{-}{ }42987.50 \cdot {\text{t }}\left[ {{\text{min}}} \right] + 713.90 \cdot {\text{Qb }}\left[ {{\text{mL}}/{\text{min}}} \right] \cdot {\text{t }}\left[ {{\text{min}}} \right] + \varphi_{E}$$	(42)
$$C5b9 = - 431.49 - 380.40 \cdot {\text{K}}\left[ {{\text{kcal}}/{\text{mol}}} \right] - 79.40 \cdot {\text{Qb}}\left[ {{\text{mL}}/{\text{min}}} \right] - 18.27 \cdot {\text{t}}\left[ {{\text{min}}} \right] - 14.87 \cdot {\text{K }}\left[ {{\text{kcal}}/{\text{mol}}} \right] \cdot {\text{Qb }}\left[ {{\text{mL}}/{\text{min}}} \right] + 0.22 \cdot {\text{Qb}}\left[ {{\text{mL}}/{\text{min}}} \right] \cdot {\text{t}}\left[ {{\text{min}}} \right] + \varphi_{E}$$	(43)
$$Serpin = 1.59{\text{E}}^{8} + 4.58{\text{E}}^{5} \cdot {\text{Qb }}\left[ {{\text{mL}}/{\text{min}}} \right] - 1.78{\text{E}}^{6} \cdot {\text{t }}\left[ {{\text{min}}} \right]{-}17722.05 \cdot {\text{Qb }}\left[ {{\text{mL}}/{\text{min}}} \right] \cdot {\text{t }}\left[ {{\text{min}}} \right] + \varphi_{E}$$	(44)
$$IL1\beta = 3.20 + 0.069 \cdot {\text{K }}\left[ {{\text{kcal}}/{\text{mol}}} \right] - 0.00082 \cdot {\text{Qb }}\left[ {{\text{mL}}/{\text{min}}} \right] - 0.009472 \cdot {\text{t }}\left[ {{\text{min}}} \right] + { }0.000052{ } \cdot {\text{Qb }}\left[ {{\text{mL}}/{\text{min}}} \right]{ } \cdot {\text{t }}\left[ {{\text{min}}} \right] + \varphi_{E}$$	(45)
$$IL1\alpha = 1.49 + 0.025 \cdot {\text{K }}\left[ {{\text{kcal}}/{\text{mol}}} \right] - 0.0017 \cdot {\text{Qb }}\left[ {{\text{mL}}/{\text{min}}} \right] + 0.17 \cdot {\text{t }}\left[ {{\text{min}}} \right] + 0.026 \cdot {\text{K }}\left[ {{\text{kcal}}/{\text{mol}}} \right] \cdot {\text{t }}\left[ {{\text{min}}} \right] - 7.9{\text{E}}^{5} \cdot {\text{Qb }}\left[ {{\text{mL}}/{\text{min}}} \right] \cdot {\text{t }}\left[ {{\text{min}}} \right] + \varphi_{E}$$	(46)
$$IL6 = 1.44 - 0.0071 \cdot {\text{K }}\left[ {{\text{kcal}}/{\text{mol}}} \right] + 0.023 \cdot {\text{Qb }}\left[ {{\text{mL}}/{\text{min}}} \right] - 0.0073 \cdot {\text{t }}\left[ {{\text{min}}} \right] + 0.0033 \cdot {\text{K }}\left[ {{\text{kcal}}/{\text{mol}}} \right] \cdot {\text{Qb }}\left[ {{\text{mL}}/{\text{min}}} \right]{ } + \varphi_{E}$$	(47)
$$vWF = - 495.72 - 165.44 \cdot {\text{K }}\left[ {{\text{kcal}}/{\text{mol}}} \right] + 1.48 \cdot {\text{Qb }}\left[ {{\text{mL}}/{\text{min}}} \right]{ } + \varphi_{E}$$	(48)

**Box 7:** Expanded model for different material properties for the concentrations of biomarkers as a function of membrane affinity to fibrinogen (K), Qb, and t, where Qb = 0 and t = 30 min.

$$C5a = 1.074{\text{E}}^{5} - 8533.82 \cdot {\text{K }}\left[ {{\text{kcal}}/{\text{mol}}} \right] + 102.68 \cdot {\text{Qb }}\left[ {{\text{mL}}/{\text{min}}} \right] - 304.16 \cdot {\text{t }}\left[ {{\text{min}}} \right] + \varphi_{M}$$	(49)
$$Properdin = 1.12{\text{E}}^{7} + 7680.83 \cdot {\text{Qb }}\left[ {{\text{mL}}/{\text{min}}} \right]{ }{-}{ }42987.50 \cdot {\text{t }}\left[ {{\text{min}}} \right] + 713.90 \cdot {\text{Qb }}\left[ {{\text{mL}}/{\text{min}}} \right] \cdot {\text{t }}\left[ {{\text{min}}} \right] + \varphi_{M}$$	(50)
$$C5b9 = - 431.49 - 380.40 \cdot {\text{K}}\left[ {{\text{kcal}}/{\text{mol}}} \right] - 79.40 \cdot {\text{Qb}}\left[ {{\text{mL}}/{\text{min}}} \right] - 18.27 \cdot {\text{t}}\left[ {{\text{min}}} \right] - 14.87 \cdot {\text{K }}\left[ {{\text{kcal}}/{\text{mol}}} \right] \cdot {\text{Qb }}\left[ {{\text{mL}}/{\text{min}}} \right] + 0.22 \cdot {\text{Qb}}\left[ {{\text{mL}}/{\text{min}}} \right] \cdot {\text{t}}\left[ {{\text{min}}} \right] + \varphi_{M}$$	(51)
$$Serpin = 1.59{\text{E}}^{8} + 4.58{\text{E}}^{5} \cdot {\text{Qb }}\left[ {{\text{mL}}/{\text{min}}} \right] - 1.78{\text{E}}^{6} \cdot {\text{t }}\left[ {{\text{min}}} \right]{-}17722.05 \cdot {\text{Qb }}\left[ {{\text{mL}}/{\text{min}}} \right] \cdot {\text{t }}\left[ {{\text{min}}} \right] + \varphi_{M}$$	(52)
$$IL1\beta = 3.20 + 0.069 \cdot {\text{K }}\left[ {{\text{kcal}}/{\text{mol}}} \right] - 0.00082 \cdot {\text{Qb }}\left[ {{\text{mL}}/{\text{min}}} \right] - 0.009472{ } \cdot {\text{t }}\left[ {{\text{min}}} \right] + { }0.000052{ } \cdot {\text{Qb }}\left[ {{\text{mL}}/{\text{min}}} \right]{ } \cdot {\text{t }}\left[ {{\text{min}}} \right] + \varphi_{M}$$	(53)
$$IL1\alpha = 1.49 + 0.025 \cdot {\text{K }}\left[ {{\text{kcal}}/{\text{mol}}} \right] - 0.0017 \cdot {\text{Qb }}\left[ {{\text{mL}}/{\text{min}}} \right] + 0.17 \cdot {\text{t }}\left[ {{\text{min}}} \right] + 0.026 \cdot {\text{K }}\left[ {{\text{kcal}}/{\text{mol}}} \right] \cdot {\text{t }}\left[ {{\text{min}}} \right] - 7.9{\text{E}}^{5} \cdot {\text{Qb }}\left[ {{\text{mL}}/{\text{min}}} \right] \cdot {\text{t }}\left[ {{\text{min}}} \right] + \varphi_{M}$$	(54)
$$IL6 = 1.44 - 0.0071 \cdot {\text{K }}\left[ {{\text{kcal}}/{\text{mol}}} \right] + 0.023 \cdot {\text{Qb }}\left[ {{\text{mL}}/{\text{min}}} \right] - 0.0073 \cdot {\text{t }}\left[ {{\text{min}}} \right] + 0.0033 \cdot {\text{K }}\left[ {{\text{kcal}}/{\text{mol}}} \right] \cdot {\text{Qb }}\left[ {{\text{mL}}/{\text{min}}} \right]{ } + \varphi_{M}$$	(55)
$$vWF = - 495.72 - 165.44 \cdot {\text{K }}\left[ {{\text{kcal}}/{\text{mol}}} \right] + 1.48 \cdot {\text{Qb }}\left[ {{\text{mL}}/{\text{min}}} \right]{ } + \varphi_{M}$$	(56)

### In vitro incubation of membrane materials in HD patient uremic serum

**Figure 4:**
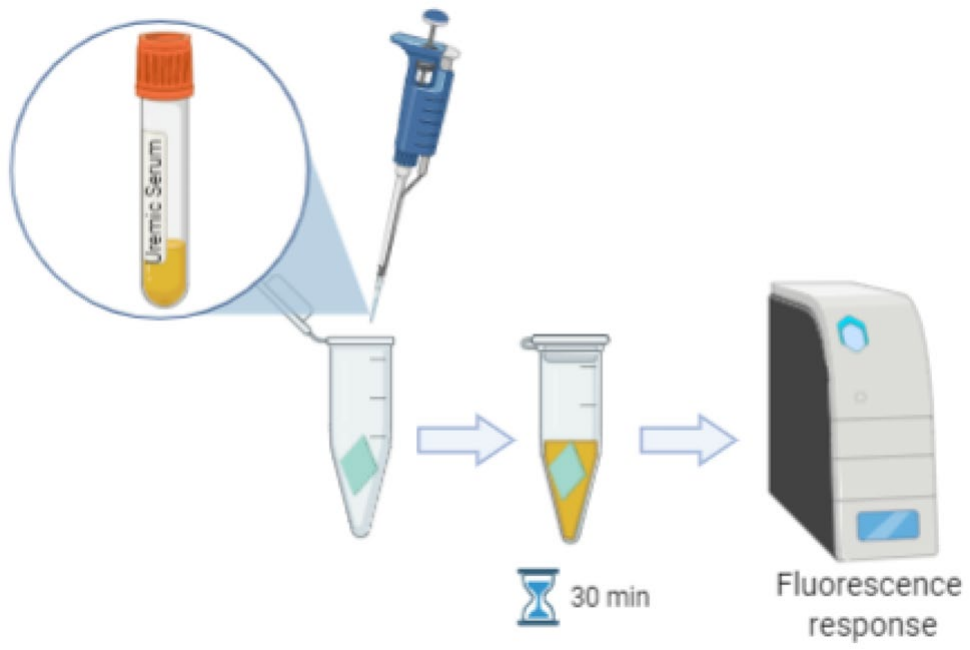
Illustration of uremic serum incubation with different membrane materials. The serum collected from HD patients was added to an Eppendorf tube containing a small sample of membrane material (in green). The interaction took place for 30 min after which the membrane sample was removed and the serum analyzed based on fluorescence response (BioRender—GP22PBN2KN).

In vitro incubation experiments were performed to assess the interaction between uremic serum and various membrane materials in the absence of hydrodynamic conditions. As illustrated in Fig. 4, small samples of the membrane materials were incubated in ~ 100 µL serum samples from HD patients collected prior to their dialysis session. After 30 min, the membrane was transferred to a separate previously unused tube. Next, a 1-µl aliquot of the corresponding serum sample was prepared and subjected to Luminex and ELISA assays.

### Theoretical computation of interaction affinity using molecular docking

The 1-phenoxy 4-(phenylsulfonyl) benzene and (2S,3S,5S,6S)-6-(acetoxymethyl)-2-(((2S,4S,5S)-2,3-diacetoxy-5-(acetoxymethyl)-6-methoxytetrahydro-2H-pyran-4-yl)oxy)-5 methoxytetrahydro-2H-pyran-3,4-diyl diacetate were selected as monomer models (ligands) for the PAES and CTA membranes, respectively. The structures were drawn in Chemdraw software and the Chemdraw format of the ligand then converted to .pdb format (Fig. S3a, first and second rows). Energy minimization was performed for the structure using Chem3D Ultra (Version 8.0) software (Fig. S3b, first and second rows).

Docking studies were carried out using AutoDock software version 4.0 to determine favorable structural characteristics for protein–ligand interactions, as described in our previous study^20^. The three-dimensional X-ray structures of FB (PDB code: 2vdm) were chosen as templates for the modeling study. Water molecules were removed from the protein, Kollman charges were added, nonpolar hydrogens were merged, and, finally, AutoDock 4 atom types were assigned to achieve the PDBQT format of the protein structure. The model was created and minimized using HyperChem 8.0 and then converted to PDBQT file format with AutoDock tools. The active site was defined as a grid box around the crystallographic ligand and the interactions were analyzed, as explained in our previous studies^20,24^.

To expand our investigation and attempt to develop a biocompatibility model for a range of membrane materials based on the predicted affinity to FB obtained via molecular docking, we performed a similar study using ligands corresponding to polyacrylonitrile (PAN), polyvinylidene fluoride (PVDF), and a PVDF-coated novel zwitterionic (ZW) material (PVDF-ZW), as presented in Fig. S3 (third to fifth row). The affinity to human serum FB was obtained for all membrane materials (Table S2).

### Theoretical investigations and modeling of inflammatory biomarker responses

**Figure 5:**
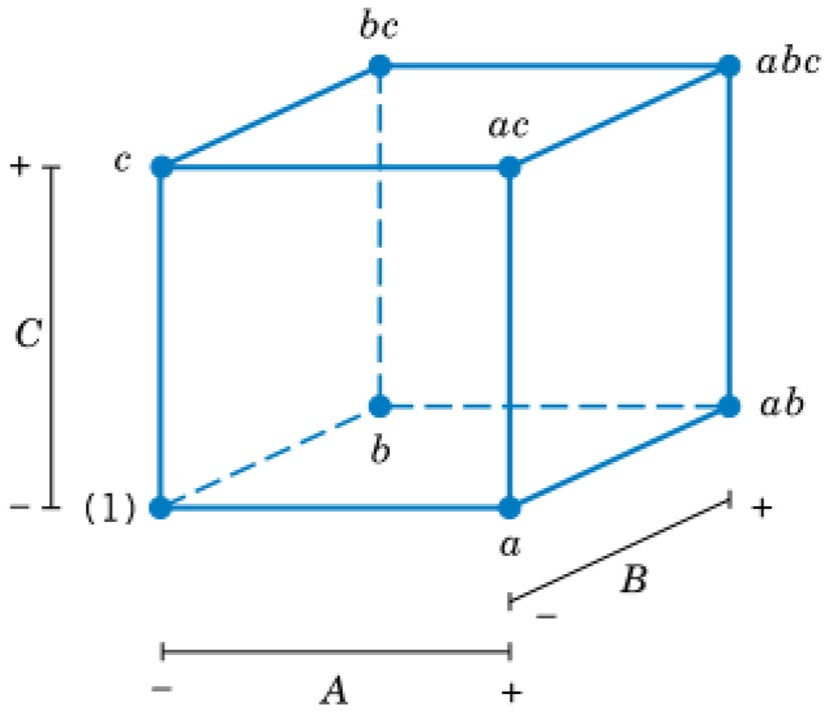
Geometric representation of the 2^3^ experimental design.

Multiple 2^k^ (k = 3) factorial designs were used to analyze the data collected from HD patients and incubated blood samples. 2^3^ factorial designs are simple statistical experimental designs that provide valuable information regarding the main effects and two-factor interactions using the smallest number of experiments possible. This approach was chosen to avoid the sparsity effect observed in higher-order designs^47^. Each 2^3^ design has eight treatment combinations, as illustrated in Fig. 5, where each corner of the cube represents one treatment combination tested. Three factors were selected for each model (A, B, and C), where the A was one of the membrane properties, and B and C were Qb (mL/min) and t (min), respectively. The membrane properties were divided into three groups: membrane morphology, chemistry, and theoretical affinity to FB. Membrane morphology was characterized in terms of pore size (Dp, nm), roughness (Ra, nm), and zeta potential (ζ, mV); membrane chemistry in terms of the percentage of sulfur (%S); and membrane tendency to protein adsorption in terms of affinity (K, kcal/mol) to FB. The response variables selected were the measured concentration (pg/mL) of the eight biomarkers (serpin/antithrombin-III, properdin, C5a, IL-1α, IL-1β, IL-6, vWF, and C5b-9).

**Figure 6:**
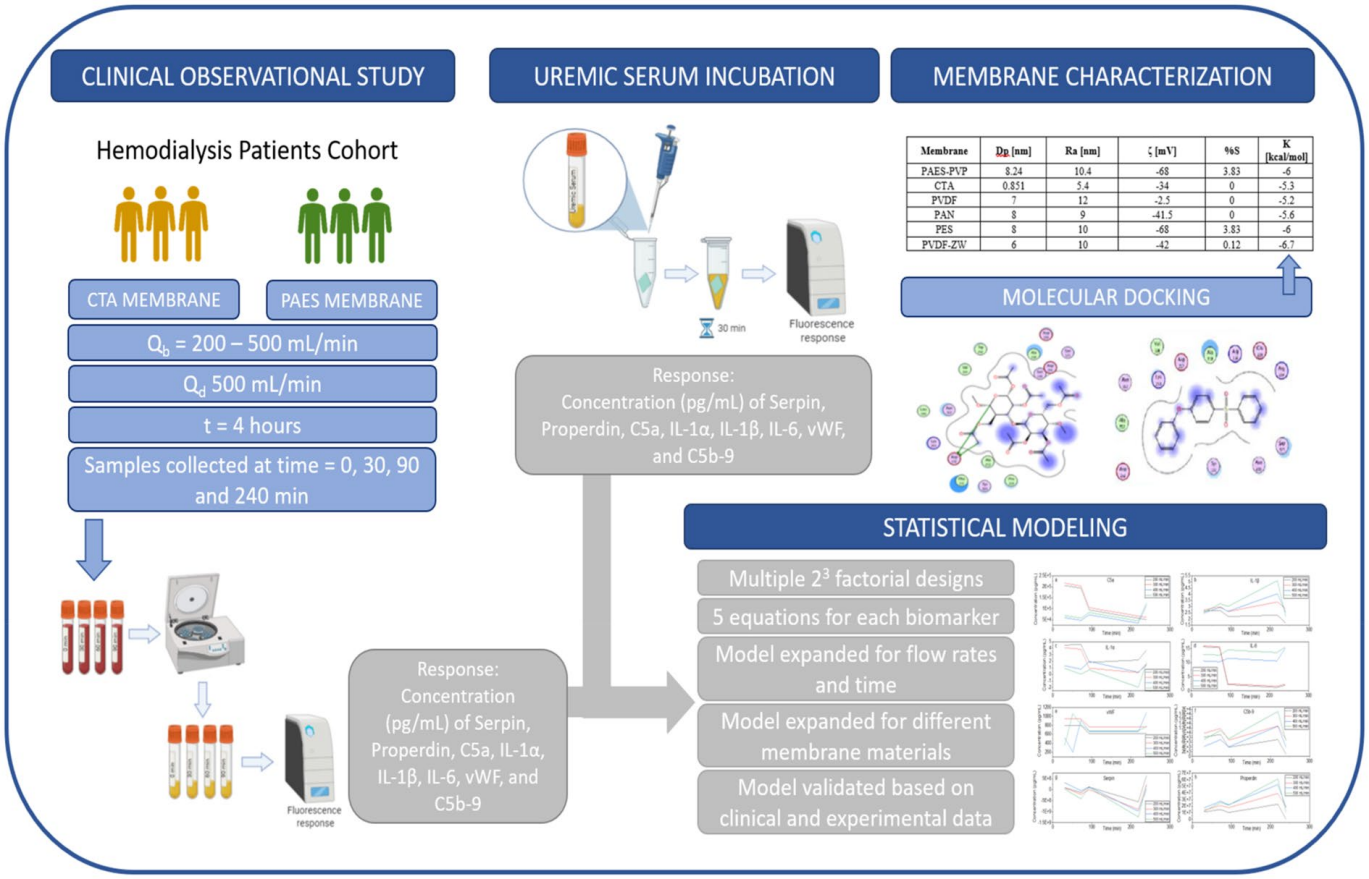
Summary of the modeling approach to predict the inflammatory biomarkers (BioRender -DI22PBRTL9).

The effect of a factor corresponds to the change in the response variable produced by a change in the level of the factor. If an interaction between two factors exists, the difference in response between the level of one factor is not the same at all levels of the other factor^47^. Table S8 shows the factors that correspond to each independent variable as well as the values corresponding to low and high levels. DesignExpert software was utilized to perform the calculations and obtain the models, as summarized in Fig. 6. The least-squares method was applied in a hierarchical fashion to compute the coefficients, and the software applied a forward selection method to calculate the effects. The significance of the coefficients was assessed by an F-test and the p-value was used to set the level of confidence of the F-test. A p-value of less than 0.05 indicates the model term is significant. Marginally significant or insignificant terms were included when necessary to maintain the model hierarchy.

### Research ethical principles of human experimentation

Dr. Amira Abdelrasoul, the principal investigator of the project, obtained University of Saskatchewan Research Ethics Approval as well as Saskatchewan Health Authority Operational Approval to conduct the research in Saskatchewan, Canada. All experimental protocols involving humans were conducted according to the governing law. All study participants from St. Paul's Hospital signed a written informed consent, approved by the University of Saskatchewan Biomedical Research Ethics Board.

## Supplementary Information

Supplementary Information.

## Data Availability

The raw/processed data required to reproduce these findings cannot be shared at this time, as the data are critical to ongoing research.
