# Supplementary material for: Hemodialysis biocompatibility mathematical models to predict the inflammatory biomarkers released in dialysis patients based on hemodialysis membrane characteristics and clinical practices
Source: Sci Rep. 2021 Nov 29;11:23080. doi: 10.1038/s41598-021-01660-1 (PMC8630185; doi:10.1038/s41598-021-01660-1)
Supplement: Supplementary file 1 — Supplementary Information. [file 41598_2021_1660_MOESM1_ESM.docx]

**Hemodialysis Biocompatibility Mathematical Models to predict the Inflammatory Biomarkers Released in Dialysis Patients based on Hemodialysis Membrane Characteristics and Clinical Practices**

**Amira Abdelrasoul ^1,2*^, Heloisa Westphalen^1^, Shaghayegh Saadati^1,2^, Ahmed Shoker^3,4^**

*^1^Department of Chemical and Biological Engineering, University of Saskatchewan, 57 Campus Drive, Saskatoon, Saskatchewan, S7N 5A9, Canada.*

*^2^ Division of Biomedical Engineering, University of Saskatchewan, 57 Campus Drive, Saskatoon, Saskatchewan, S7N 5A9, Canada.*

*^3^ Nephrology Division, College of Medicine, University of Saskatchewan, 107 Wiggins Rd, Saskatoon, SK S7N 5E5*

*^4^ Saskatchewan Transplant Program, St. Paul's Hospital, 1702 20th Street West Saskatoon Saskatchewan S7M 0Z9 Canada*

****Corresponding Author: amira.abdelrasoul@usask.ca, Tel: (306) 966 2946, Fax: (306) 966 4777***

**SUPPORTING INFORMATION**


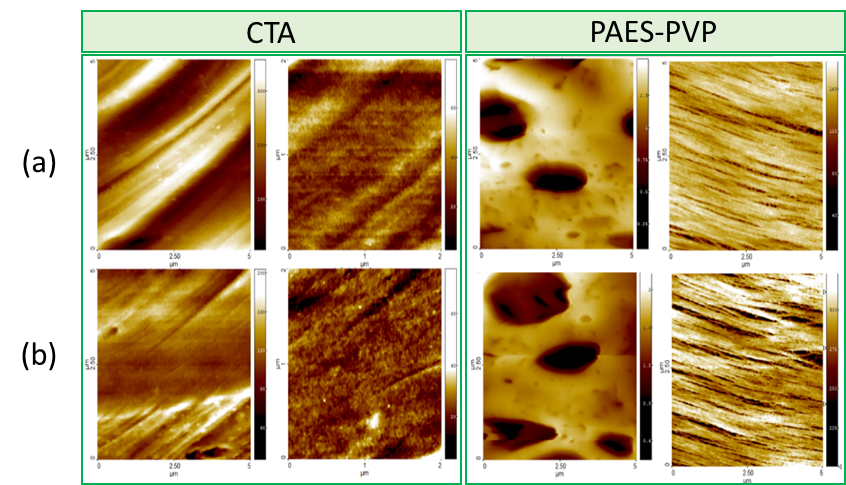


**Figure S1.** AFM micrographs of pristine CTA and PAES HD membrane fibers collected for two different spots in close proximity (a and b, first second rows); micrographs of the inner and outer surfaces of the polymer membrane fibers are placed in the first and second column


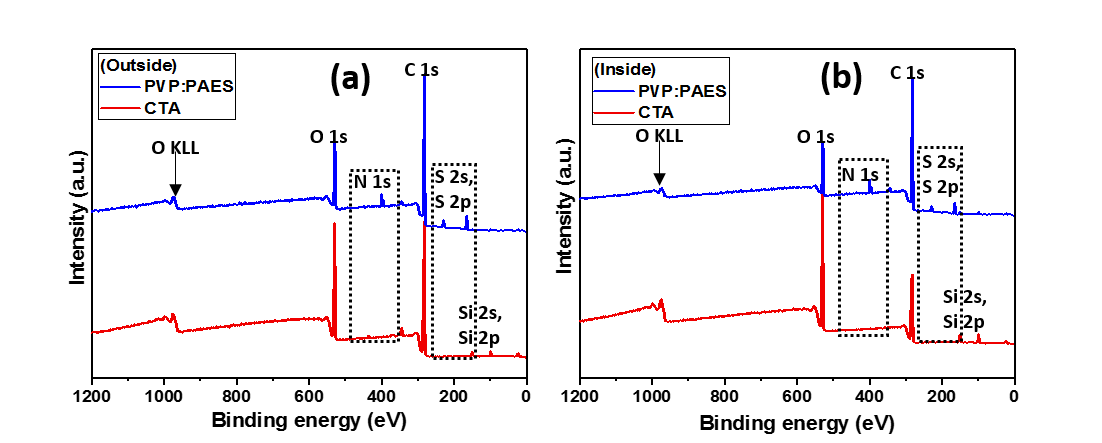


**Figure S2**. XPS wide-scan spectra of the outside (a) and inside (b) of CTA and PAES polymer membrane fibers (CasaXPS ,Version 2.3.24, www.casaxps.com/)


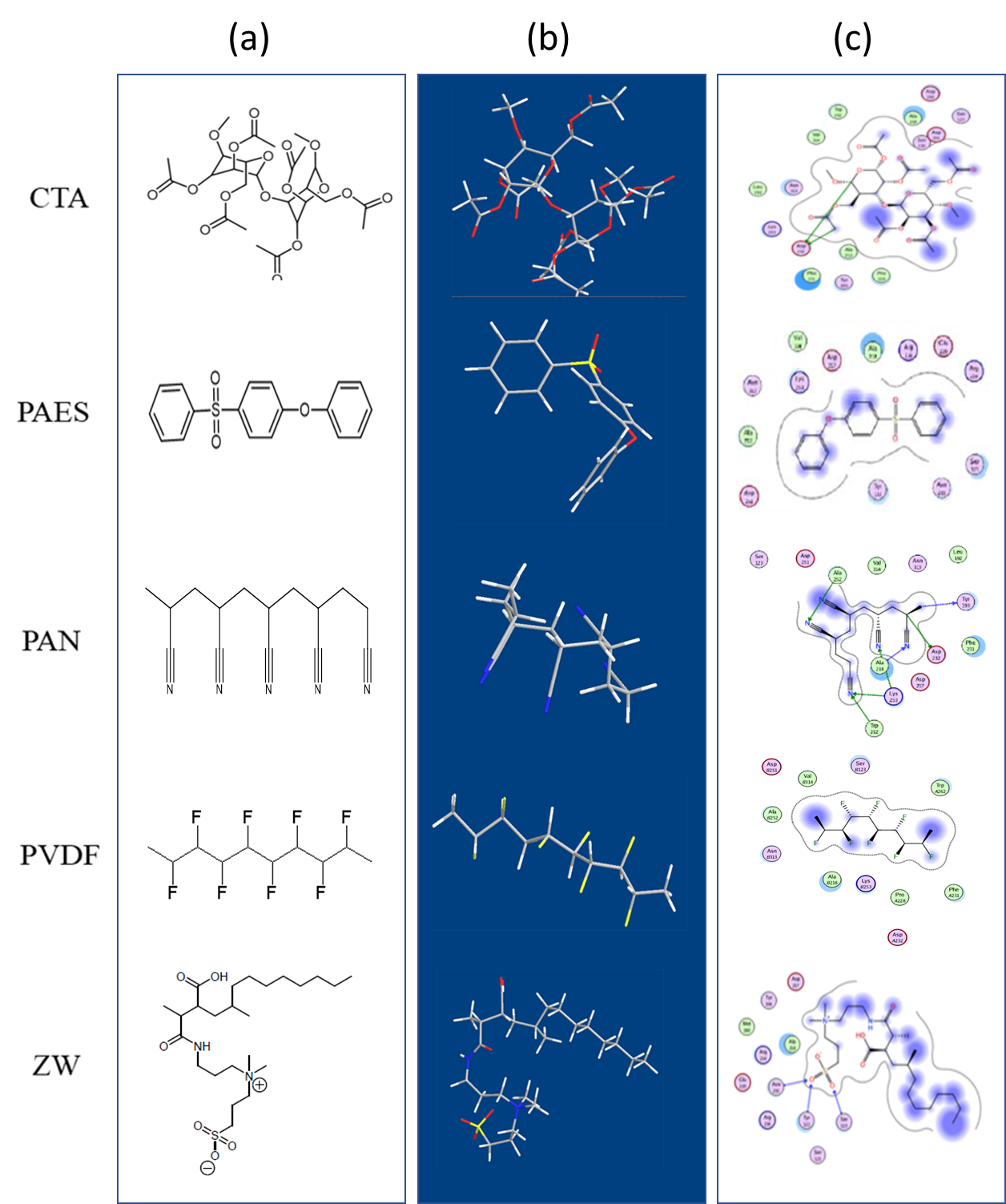


**Figure S3.** **(a)** Chemical structure of **CTA:** (2S,3S,5S,6S)-6-(acetoxymethyl)-2-(((2S,4S,5S)-2,3-diacetoxy-5-(acetoxymethyl)-6-methoxytetrahydro-2H-pyran-4-yl)oxy)-5 methoxytetrahydro-2H-pyran-3,4-diyl diacetate; **PAES:** 1-phenoxy 4-(phenylsulfonyl) benzene; **PAN:** (C_15_H_17_N_5_) decane-1,3,5,7,9-pentacarbonitrile; **PVDF:** 2,3,4,5,6,7,8,9-octafluorodecane (C_10_H_14_F_8_); and **PVDF-ZW:** 3-((3-(3-carboxy-2,5-dimethyltridecanamido) propyl) dimethylammonio) propane-1-sulfonate (C_24_H_48_N_2_O_6_S) (Chemdraw software; version 14, www.chemdraw.com/) **(b)** Respective optimized structure of the ligand molecules (Chem3D Ultra; Version 8.0, www.chemdraw.com/) **(c)** 2D molecular docking image of each ligand interacting with fibrinogen molecule (Molecular Operating Environment (MOE), MOE 2014.0901, www.chemcomp.com)


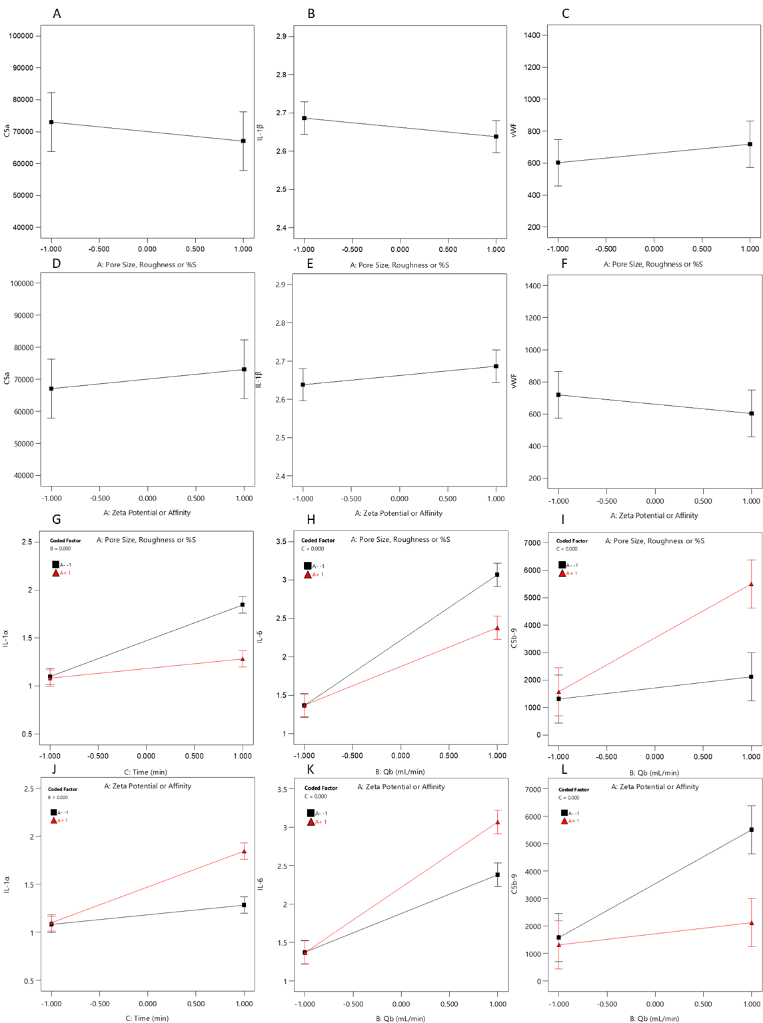


**Figure S4**. Main effects and interactions for all models *(*DesignExpert, Version 12.0.12.0, <https://www.statease.com/software/design-expert/>)

**Tables**

**Table S1**. Fit statistics

| **Biomarker** | **Equation #** | **R^2^** | **Adequate Precision** |
| --- | --- | --- | --- |
| C5a | 1,9,17,25,33 | 0.8591 | 6.94 |
| IL-1β | 2,10,18,26,34 | 0.966 | 11.18 |
| IL-1α | 3,11,19,27,35 | 0.9988 | 52.28 |
| IL-6 | 4,12,20,28,36 | 0.9935 | 25.24 |
| vWF | 5,13,21,29,37 | 0.7669 | 5.71 |
| Serpin | 6,14,22,30,38 | 0.8209 | 5.51 |
| Properdin | 7,15,23,31,39 | 0.8741 | 6.90 |
| C5b-9 | 8,16,24,32,40 | 0.987 | 14.73 |

**Table S2**. Affinity values for ligand-fibrinogen interactions determined from molecular docking studies

| Ligand | *K* affinity to FB  (Kcal mol^-1^) |
| --- | --- |
| PAES | -6.00 |
| CTA | -5.3 |
| PAN | -5.6 |
| PVDF | -5.2 |
| PES-ZW | -6.7 |
| PVDF-ZW | -6.7 |

**Table S3.** Membrane properties

| **Membrane** | **Dp [nm]** | **Ra [nm]** | **ζ [mV]** | **%S** | **K [kcal/mol]** |
| --- | --- | --- | --- | --- | --- |
| PAES-PVP | 8.24 | 10.4 | -68 | 3.83 | -6 |
| CTA | 0.851 | 5.4 | -34 | 0 | -5.3 |
| PVDF | 7 | 12 | -2.5 | 0 | -5.2 |
| PAN | 8 | 9 | -41.5 | 0 | -5.6 |
| PES | 8 | 10 | -68 | 3.83 | -6 |
| PVDF-ZW | 6 | 10 | -42 | 0.12 | -6.7 |

**Table S4.** Surface roughness measurements for hollow polymer membrane fibers.

| Parameter | CTA | | PAES-PVP | |
| --- | --- | --- | --- | --- |
|  | Inside  (2 × 2 µm)  (n = 3) | Outside  (2 × 2 µm)  (n = 4) | Inside  (2 × 2 µm)  (n = 3) | Outside  (1.5 × 1.5 µm)  (n = 4) |
| R_a_ (nm) | 5.4 ± 1.9 | 5.4 ± 1.9 | 10.4 ± 4.0 | 10.5 ± 5.0 |
| R_RMS_ (nm) | 7.5 ± 3.0 | 7.5 ± 3.0 | 15.3 ± 5.9 | 15.4 ± 6.8 |

**Table S5.** Percent (%) abundance of elements within the membrane fibers

| Fiber | Morphology | Element (%) | | | | | | |
| --- | --- | --- | --- | --- | --- | --- | --- | --- |
|  |  | O 1s | C 1s | Si 2 p | Ca 2p | N 1s | S 2p | B 1s |
| CTA | Inside | 29.30 | 64.37 | 6.33 | — | — | — | — |
|  | Outside | 21.97 | 74.51 | 2.50 | 1.02 | — | — | — |
| PAES-PVP | Inside | 12.29 | 75.60 | 1.60 | — | 2.08 | 3.83 | 4.60 |
|  | Outside | 13.56 | 76.47 | 0.96 | — | 1.97 | 4.39 | 2.65 |

**Table S6.** BET surface area and zeta potential values for CTA and PAES-PVP polymer membrane fibers

| Fiber | Zeta potential (mV) | Pore size (nm) | BET surface area (m^2^ g^-1^) |
| --- | --- | --- | --- |
| CTA | –34 | 0.851 | 14.47 ± 1.48 |
| PAES:PVP | –68 | 8.24 | 1.99 ± 0.32 |

**Table S7.** Basic HD patient information

|  |  | Healthy Controls | | Hemodialysis Patients | |
| --- | --- | --- | --- | --- | --- |
| Variables | **Values** | **N** | **%** | **N** | **%** |
| Gender | Female | 1 | 50 | 5 | 41.66 |
|  | Male | 1 | 50 | 7 | 58.33 |
| Age | <50 years | 0 | 0 | 5 | 41.66 |
|  | ≥50 years | 2 | 100 | 7 | 58.33 |
| Race | Non-black | 2 | 100 | 12 | 100 |
|  | Black | 0 | 0 | 0 | 0 |
| BMI^a^ | <27 kg/m^2^ | 0 | 0 | 0 | 0 |
|  | ≥27 kg/m^2^ | 2 | 100 | 12 | 100 |
| CAD^b^ | Yes | 0 | 0 | 5 | 41.66 |
|  | No | 2 | 100 | 7 | 58.33 |
| Diabetes | Yes | 0 | 0 | 6 | 50.00 |
|  | No | 2 | 100 | 6 | 50.00 |
| Hypertension | Yes | 1 | 50 | 10 | 83.33 |
|  | No | 1 | 50 | 2 | 16.66 |
| PVD^c^ | Yes | 0 | 0 | 4 | 33.33 |
|  | No | 2 | 100 | 8 | 66.67 |
| ESRD duration | <1 year | NA | NA | 1 | 8.33 |
|  | 1–5 years |  |  | 10 | 83.34 |
|  | >5 years |  |  | 1 | 8.33 |

BMI^a^: [body mass index](https://www.sciencedirect.com/topics/medicine-and-dentistry/body-mass-index) (kg/m^2^); CAD^b^: [coronary artery disease](https://www.sciencedirect.com/topics/medicine-and-dentistry/coronary-artery-disease); PVD^C^: [peripheral vascular disease](https://www.sciencedirect.com/topics/medicine-and-dentistry/peripheral-vascular-disease).

**Table S8.** Parameter levels used in the statistical modeling based on membrane properties

| **Factor** | **Variable** | **Level** | | **Unit** |
| --- | --- | --- | --- | --- |
|  |  | **Low** | **High** |  |
|  |  | **(-1)** | **(+1)** |  |
| A | Pore Size (Dp) | 0.851 | 8.24 | nm |
| A | Roughness (Ra) | 5.4 | 10.4 | nm |
| A | Zeta Potential (ζ) | -68 | -34 | mV |
| A | Sulfur content (%S) | 0 | 3.83 | % |
| A | Affinity (K) | -6 | -5.3 | kcal/mol |
| B | Blood flow rate (Qb) | 0 | 300 | mL/min |
| C | Treatment Time (t) | 0 | 30 | min |

**Nomenclature**

| AA | Arachidonic acid |
| --- | --- |
| AFM | Atomic force microscopy |
| ADP | Adenosine 5'-diphosphate |
| AKI | Acute kidney injury |
| BET | Brunauer-Emmett-Teller |
| CKD | Chronic kidney disease |
| CRP | C-reactive protein |
| CTA | Cellulose triacetate |
| C3 | Complement component 3 |
| C5a | Complement component 5a |
| C5b-9 | Terminal complement complex |
| Dp | Pore size (nm) |
| FB | Fibrinogen |
| HD | Hemodialysis |
| IL | Interleukin |
| K | Affinity |
| MDS | Molecular dynamic simulations |
| PAES-PVP | Polyvinylpyrrolidone:polyarylethersulfone |
| PAN | Polyacrylonitrile |
| PVDF | Polyvinylidene fluoride |
| Qb | Blood flow rate (mL/min) |
| Ra | Average roughness (nm) |
| RBC | Red blood cells |
| ROS | Reactive oxygen species |
| RRMS | Root mean square roughness |
| RRTs | Renal replacements therapies |
| t | Treatment time (min) |
| TNF | Tumor necrosis factor |
| TXA2 | Thromboxane A2 |
| vWF | Von Willebrand factor |
| XPS | X-ray photoelectron spectroscopy |
| ZW | Zwitterionic |
| 5-LO | 5-Lipoxygenase |
| ζ | Zeta potential (mV) |
| $\varphi_{E}$ | Hydrodynamic correction factor |
| $\varphi_{M}$ | Material correction factor |
| %S | Percentage of sulfur |
